# RNA binding candidates for human ADAR3 from substrates of a gain of function mutant expressed in neuronal cells

**DOI:** 10.1093/nar/gkz815

**Published:** 2019-09-25

**Authors:** Yuru Wang, Dong hee Chung, Leanna R Monteleone, Jie Li, Yao Chiang, Michael D Toney, Peter A Beal

**Affiliations:** Department of Chemistry, University of California, One Shields Ave, Davis, CA 95616, USA

## Abstract

Human ADAR3 is a catalytically inactive member of the Adenosine Deaminase Acting on RNA (ADAR) protein family, whose active members catalyze A-to-I RNA editing in metazoans. Until now, the reasons for the catalytic incapability of ADAR3 has not been defined and its biological function rarely explored. Yet, its exclusive expression in the brain and involvement in learning and memory suggest a central role in the nervous system. Here we describe the engineering of a catalytically active ADAR3 enzyme using a combination of computational design and functional screening. Five mutations (A389V, V485I, E527Q, Q549R and Q733D) engender RNA deaminase in human ADAR3. By way of its catalytic activity, the ADAR3 pentamutant was used to identify potential binding targets for wild type ADAR3 in a human glioblastoma cell line. Novel ADAR3 binding sites discovered in this manner include the 3′-UTRs of the mRNAs encoding early growth response 1 (EGR1) and dual specificity phosphatase 1 (DUSP1); both known to be activity-dependent immediate early genes that respond to stimuli in the brain. Further studies reveal that the wild type ADAR3 protein can regulate transcript levels for DUSP1 and EGR1, suggesting a novel role ADAR3 may play in brain function.

## INTRODUCTION

Adenosine deaminases that act on RNA (ADARs) catalyze adenosine-to-inosine (A-to-I) editing in RNA (1), an essential post-transcriptional modification for normal metazoan life and believed to contribute to protein diversity, regulate RNA processing and act as a blockade of auto-immune response in higher organisms (2–4). Malfunctions in ADARs are associated with human diseases, including Aicardi-Goutieres Syndrome (AGS), Dyschromatosis symmetrica hereditaria (DSH) and cancers (5–7). Three members have been identified in the human ADAR family; ADAR1, ADAR2 and ADAR3 (1). ADARs are composed of double-stranded RNA binding domains (dsRBDs) that facilitate substrate recognition and a C-terminal catalytic (deaminase) domain that catalyzes the A-to-I deamination (1). Uniquely, ADAR1 also harbors Z-binding domains which facilitate binding to Z-conformation duplexes and ADAR3 has an N-terminal arginine-rich domain which enables it to bind to single stranded RNA (8,9). ADARs deaminate adenosines within duplex RNAs using a base-flipping mechanism (10).

Unlike ADAR1 and ADAR2, which are ubiquitously expressed and catalyze deamination at thousands of sites in the human transcriptome, ADAR3 is exclusively expressed in the brain and catalytic activity has not been observed (11). Puzzlingly, the ADAR3 deaminase domain contains key residues known to be important for the catalysis of adenosine deamination (i.e. histidine and cysteine residues that coordinate the catalytic zinc ion and a glutamate residue that facilitates proton transfers) in ADAR1 and ADAR2, and shares high sequence similarity to the ADAR2 deaminase domain (50% identity and 70% similarity) (6,8). Although it is widely accepted that ADAR3 is catalytically inactive, the reason for its lack of catalytic activity is not clear. It has been suggested that the absence of ADAR3 activity is a result of its inability to dimerize (12,13). There is also the intriguing possibility that ADAR3 targets and edits (or simply binds to) yet unidentified RNA substrates in the brain (11,14).

While the studies for ADAR1 and ADAR2 have been blooming over the years, those for ADAR3 are relatively rare. However, the exclusive expression of ADAR3 in the brain, which is the most highly edited organ in the adult mouse, suggests a unique function for ADAR3 in the nervous system (11). Indeed, Mladenova *et al.* recently reported that ADAR3 is involved in memory and learning in mice, demonstrating a central role in the mammalian nervous system (15); yet, the underlying molecular mechanism is unclear.

It has been suggested that ADAR3 regulates the activity of ADAR1 and ADAR2 (8,16,17). For instance, *in vitro* studies have shown that ADAR3 can inhibit editing on the 5-HT_2c_R RNA by ADAR1 and ADAR2 (8). In addition, a recent study showed that ADAR3 can regulate editing of the GluR B receptor mRNA *in vivo*, via its dsRBDs but not its deaminase domain (17). The inhibitory role of ADAR3 on the editing activities of ADAR1 and ADAR2 was further demonstrated by a recent study analyzing the overall editing levels in relation to ADAR3 expression in various brain tissues (16). Yet additional specific transcripts that are competitively bound by ADAR3 are unknown, limiting our understanding of the biological processes in which ADAR3 may be involved as a binding competitor of the other ADARs.

On the other hand, ADAR3 may have a direct effect on the fate of cellular RNAs as an RNA binding protein, independent of RNA-editing. Both ADAR1 and ADAR2 are involved in important biological processes independent of their A-to-I editing functionality (18,19). Indeed, human ADAR3 (hADAR3) has been shown to interact with the GGGGCC expanded repeat in the endogenous C9ORF72 RNA and the interaction appears to play a role in the intranuclear GGGGCC_exp_ RNA foci formation (20). The identification of additional cellular binding targets is required to fully define ADAR3′s biological functions.

Here, in order to elucidate the reasons for the catalytic incapability of ADAR3 and further provide insights into its biological role, we carried out targeted mutagenesis to generate a catalytically active ADAR3 and used the active mutant to identify potential binding targets of the wild type human ADAR3 protein. Combining computational design using the Janus program (21,22) and functional screening of ADAR2/ADAR3 chimeras, we enabled human ADAR3 as an active deaminase by introducing five point mutations in its deaminase domain. These results show that select, catalytically critical residues, which had not previously been appreciated, are altered in ADAR3. We show that the active mutant efficiently deaminates RNA substrates both *in vitro* and *in vivo*. Taking advantage of the capacity of the active mutant to put inosine marks on bound substrates, expression of the active mutant in the human glioblastoma cell line U87, followed by whole transcriptome sequencing, identified potential cellular binding targets of hADAR3. The ADAR3-interacting RNAs identified in this manner include transcripts with known ADAR1 and ADAR2 editing sites but also RNAs not previously known to interact with any ADAR. Two particularly interesting novel targets are 3′ UTRs of mRNAs of EGR1 and DUSP1, which are known to be activity-dependent immediate early genes that respond to stimuli in the brain. We have demonstrated the interaction between the wild type ADAR3 and these two mRNAs and, more importantly, that ADAR3 expression can regulate the transcript abundance of these two genes in cells. Our results suggest a novel function for ADAR3 as a direct regulator of certain IEGs expression through 3′ UTR interactions, one possible mechanism by which ADAR3 is involved in cognitive processes in mammals.

## MATERIALS AND METHODS

### General methods

Components for yeast culture media were purchased from BTS (biological grade peptone, yeast extracts and biological grade agar), BD (yeast nitrogen base w/o amino acid) and Sigma-Aldrich (glucose, glycerol, lactate, galactose, raffinose, amino acids, yeast synthetic dropout supplement medium without uracil and yeast synthetic dropout medium without tryptophan). Phusion hot start DNA polymerase was purchased from Thermo Scientific for high fidelity DNA amplification. Restriction enzymes, T4 ligase and other cloning enzymes were obtained from New England Biolabs. Ligation products were transformed into XL 10 gold cells. The sequences of plasmids were confirmed by Sanger sequencing. Plasmid extraction from *Escherichia coli* was carried out with QIAprep Spin Miniprep kit (Qiagen). Oligonucleotides were synthesized by Integrated DNA Technology. Codon-optimized ADAR3 genes were synthesized by Thermo Fisher Scientific.

### Janus prediction

ADAR1 sequences were retrieved by searching the hADAR1 full length protein sequence against the UniProtKB protein sequences database using HMMER v3.1b2. The Z-alpha domain was used as a signature to retrieve ADAR1 sequences. ADAR2 sequences present in the retrieved ADAR1 search were identified based on its 5′ binding loop sequence, and removed (23). A total of 84 ADAR1 sequences were identified. ADAR2 sequences were retrieved by searching the full length hADAR2 sequence against the UniProtKB protein sequences database using HMMER v3.1b2. Returned sequences that did not contain the 5′ binding loop motif of ADAR2 were removed. A total of 124 ADAR2 sequences were retrieved. Two methods were combined to search for ADAR3 sequences. First, a PSI-BLAST search was done using the hADAR3 full protein sequence. ADAR2 sequences were identified based on its 5′ binding loop sequence and were removed. Second, the ADAR3 full length sequence was searched against the UniProtKB protein sequences database using HMMER v3.1b2. Sequences from the two searches were combined and a total of 89 ADAR3 sequences were retrieved.

Janus predictions employed sequence alignments generated using MUSCLE, with ADAR3 sequences as the ‘start’ group, and ADAR1 and ADAR2 sequences as the ‘target’ group. A homology model of the hADAR3 catalytic domain was created using SWISS-MODEL and PDB ID:5HP2 as a template. This structure was used as the reference structure for distance calculations in Janus. Atom 683 (O2 of E434) was used for measuring distances from the active site. Janus 18v1 (see Supplementary Materials) was used for mutation predictions with the human ADAR3 protein sequence as the reference sequence.

### ADAR protein constructs

Genes encoding ADARs for expression in yeast were inserted in yeast expression plasmid YEpTOP2PGAL1. The hADAR3 gene codon-optimized for expression in yeast was synthesized by Thermo Fisher Scientific. To construct ADAR3 libraries covering all possible combinations of the top 11 mutations predicted by Janus, a PCR method named MPPCR, developed to rapidly generate multi-site-specific libraries of a gene of interest, was used (24). Specifically, mutant megaprimers were first generated in separate PCR reactions using a forward mutant primer and a reverse primer. Subsequently, these megaprimers were mixed and used in a single PCR reaction containing the ADAR3 gene template and a forward primer. Cloning was performed using standard Gibson assembly protocols and used to transform *E. coli* (DH5α) which generated a total of 5 × 10^7^ transformants. The incorporation of all 11 mutations were confirmed by sequencing analysis of the library mixture. To generate hADAR2-D/hADAR3-D chimeric proteins, a three-step PCR method was used (25). To introduce mutations to hADAR3-D, QuikChange II XL site-directed mutagenesis kit (Agilent) was used. The YEpTOP2PGAL1 yeast expression plasmid has a *GAL1* promotor, a *URA3* auxotrophic marker for selection of transformed yeast, and an ampicillin resistance marker for selection of transformed *E. coli*.

hADAR3 gene codon-optimized for expression in human cells was synthesized by Thermo Fisher Scientific and inserted into the pcDNA 3.1 vector that has a CMV enhancer and an ampicillin resistance gene for selection in *E. coli*. Point mutations were introduced into hADAR3 gene using QuikChange II XL site-directed mutagenesis kit. All constructs were confirmed by Sanger Sequencing.

### Yeast growth and ADAR overexpression in yeast

*Saccharomyces cerevisiae* INVSc1 strains containing ADAR genes were generated using a lithium acetate protocol and grown on the complete media (CM) – ura + 2% glucose plates (26). Single colonies were picked and used to inoculate 5 ml CM – ura + 2% glucose media and incubated for 24 h at 30°C with shaking at 275 rpm. Cells were pelleted from 1 ml of each culture, washed twice with ddH_2_O and used to inoculate 25 ml CM – ura + 2% raffinose + 3% galactose media for induction. The galactose cultures were grown for 48 h at 30°C with shaking at 275 rpm.

### Reporter assays

Reporter plasmids (BDF2 reporter for the colorimetric assay and yeGFP reporter for the fluorescence assay) used in this study were described previously (26,27). The reporter plasmids have a *GAL1* promotor, a *TRP1* auxotrophic marker for selection of transformed yeast, and an ampicillin resistance marker for selection of transformed *E. coli*. *Saccharomyces cerevisiae* INVSc1 strain were sequentially transformed with one of the reporters followed by plasmid expressing ADAR and plated on complete media (CM) – ura – trp + 2% glucose plate for growth. In the colorimetric assay, single colonies were transferred to plates of CM – ura – trp + 2% raffinose + 3% galactose treated with X-α-Gal. Plates were incubated in 30°C until the appearance of a green color. In the fluorescence assay, single colonies were picked from the culture plate and used to inoculate 5 ml CM – ura – trp + 2% glucose media for overnight growth. The resulting culture (0.1 ml) was used to inoculate 20 ml CM – ura – trp + 3% glycerol + 2% lactate media for another growth until the OD_600_ of the culture reaches 1–2 (∼30 h). Galactose was then added to a final concentration of 3% for induction. Cells were harvested after ∼20 h of induction and the same number of cells (10^8^) were pelleted, washed twice with PBS and measured for fluorescence in an Optiplate-96 black, black opaque 96-well microplate (PerkinElmer) using a CLARIOstar plate reader (BMG labtech), with excitation at 482/16 nm and emission at 520/10 nm.

### FACS screening of the ADAR3 mutant library

ADAR3 mutant library covering the top 11 mutations was transformed to *S. cerevisiae* INVSc1 strain already harboring the yeGFP reporter using a high efficiency lithium protocol (28). A small fraction of transformation mixture was plated on dropout plate to calculate transformation efficiency. The transformation was estimated to cover at least 50-fold of the theoretical diversity of the library. The remaining transformation mixture was used to inoculate 10 ml CM – ura – trp + 2% glucose media and grew until OD_600_ reaches to 7–8. The glucose culture was then used to inoculate CM – ura – trp + 3% glycerol + 2% lactate media (0.25 ml to 25 ml) and after an additional 36 h of growth (OD_600_ = 1–2), galactose was added to a final concentration of 3% for induction. Cells collected after 24 h induction were washed twice and resuspended with PBS to 20 000 cells/μl. Cells were sorted using Beckman Coulter Astrios EQ cell sorter at UC Davis flow cytometry shared resource laboratory. GFP excitation was at 488 nm and emission at 529/28 nm.

### *In vitro* deamination assay

hADAR3-D WT and the mutant protein M3 were overexpressed and purified from *S. cerevisiae* BCY123 strain using protocols described previously (29). Purified hADAR3 proteins were stored in 20 mM Tris–HCl, pH 8.0, 20% glycerol, 1 mM β-mercaptoethanol and 100 mM NaCl at −80°C. The 200 nt BDF200 RNA and the 150 nt GLI1 mRNA used in the deamination assays was transcribed *in vitro* and purified and refolded as described previously (26). The deamination assays were performed under single-turnover condition with 15 mM Tris–HCl PH 7.8, 41 mM potassium glutamate, 20 mM KCl, 5 mM NaCl, 0.6 mM dithiothreitol, 1.4 mM EDTA, 0.003% NP-40, 4% glycerol, 1.0 μg/ml yeast tRNA^Phe^, 160 units/ml RNAsin, 250 nM protein and 10 nM RNA. Each solution was incubated at 30°C for 30 min before adding enzyme and each reaction was allowed to proceed for different time periods at 30°C prior to stopping with either hot water or hot 1% SDS. Reacted RNAs were phenol–chloroform extracted and ethanol precipitated. RT-PCR was used to generate cDNA from deaminated RNA and the resulting cDNA were subjected to Sanger sequencing. The sequencing data were quantified using Chromas Lite (Technelysium) and ImageJ and were fit to the equation [P]*t* = α[1 – e^(-*k*obs*t)^], where [P]*t* is the fraction of deamination product at time *t*, α is the fitted reaction end point, and *k*_obs_ is the fitted rate constant using KaleidaGraph.

### MD simulation

Molecular dynamics simulations were performed with YASARA (30). The AMBER15IPQ forcefield was used (31). Simulations were setup and run using the ‘md_run’ macro provided with the program. A cubic cell extended 10 Å around the protein structure was used. Forcefield parameters for the protein bound Zn^2+^ ion were taken from the literature (32). The md_run macro fills the cell with water, calculates p*K*_a_ values for ionizable groups, neutralizes the cell, and incorporates salt as specified (NaCl at 0.15 M was used here). The cell was slowly heated to 25°C and snapshots were taken at 0.05 ns intervals over the course of 10 ns of simulation. The data after a 2 ns equilibration period were analyzed.

### Measuring editing on endogenous yeast RNA and Western blot

Following 48 h of induction, total RNAs were isolated from yeast cells (*S. cerevisiae* INVSc1 strain) overexpressing ADAR proteins using RiboPure-Yeast kit (Ambion). Editing on sites of interest was determined by amplifying approximately 200 bp cDNA surrounding the editing sites by RT-PCR. The PCR products were then subjected to Sanger sequencing. The experiments were performed in biological triplicate.

For testing expression of ADAR3 in yeast INVSc1 cells, the same number of cells (10^8^) were harvested after 48 h of induction for ADAR3 protein expression. Cells were lysed with glass beads (Sigma Aldrich) and the cell lysates were boiled and used directly for western blot analysis. The ADAR3 protein has a 10× His-tag at its N-terminus and was probed with a mouse His-probe antibody (Santa Cruz biotechnology).

### U87MG cell line culture and Western blot

Human glioblastoma cell line U87 was grown and maintained in DMEM (Gibco) supplemented with 10% FBS (Invitrogen, Carlsbad, CA, USA), 100 units/ml penicillin and 100 μg/ml streptomycin in 5% CO_2_ at 37°C. Transfection of plasmids encoding hADAR3 proteins was performed in six-well plate with lipofectamine 3000 transfection reagent (Life Technologies) when cells were grown to ∼90% confluence. Equal numbers of cells (∼10^6^) 48 h post-transfection were lysed with 125 μl native lysis buffer and cell lysate were cleared by centrifugation. The supernatants (20 μl) were used for Western blot analysis. The hADAR3 mammalian expression constructs have a HA tag and were probed with a mouse HA tag monoclonal antibody (Thermos Fisher Scientific). Actin was used as a loading control and was probed with actin antibody (Santa Cruz biotechnology).

### RNA-seq

In six-well plates, 10^6^ cells were transfected with 2.5 μg hADAR3 M3 or hADAR3 M3 E434A plasmid DNA. Total RNA was isolated from cells 48 h post-transfection using RNAqueous-4PCR kit. Strand-specific and barcode indexed RNA-seq libraries were generated from 1 μg total RNA each after poly-A enrichment using the Kapa Stranded mRNA-seq kit (Kapa Biosystems, Cape Town, South Africa) following the instructions of the manufacturer. The fragment size distribution of the libraries was verified via micro-capillary gel electrophoresis on a Bioanalyzer 2100 (Agilent, Santa Clara, CA, USA). The libraries were quantified by fluorometry on a Qubit fluorometer (Life Technologies, Carlsbad, CA, USA) and pooled in equimolar ratios. The pool was quantified by qPCR with a Kapa Library Quant kit (Kapa Biosystems) and sequenced on one lane of an Illumina HiSeq 4000 (Illumina, San Diego, CA, USA) with paired-end 100 bp reads. The experiments were performed in biological triplicate.

### RNA-seq data analysis

Raw sequencing reads were subjected to quality control: the adapter sequences were removed using scythe (https://github.com/vsbuffalo/scythe, version c128b19), and the bases that have quality score below 30 were trimmed using sickle (https://github.com/najoshi/sickle, version 7667f147e6). All reads that passed the quality control and had a minimum length of 30 bp were retained for downstream analysis. Alignment was carried out using STAR/2.5.2b (33) to the GRCh38 reference genome and the annotation from Ensembl database (34). PCR duplicates were removed using picard-tools/2.6.0 (http://broadinstitute.github.io/picard/). Single nucleotide polymorphisms (SNPs) A-to-G were identified using Freebayes/v1.1.0 (35), with the requirement of a mapping quality ≥30 and a base quality ≥30. All known SNPs were removed. In non-Alu regions, SNPs that are located within 4 base pairs of a known splice junction were removed and sites in simple repeat regions were removed. High confidence A-to-G SNPs were selected to have a minimum quality score of 60 and have no <10 reads supporting the site from all samples. High confidence A-to-G sites were tested for differential editing between control (E434A) and experiment (ADAR3 M3) using Fisher's exact test for each replicate pair. Raw *P*-values were adjusted for multiple testing using Benjamini & Hochberg method (36). The sites that have an FDR adjusted *P*-value <0.05 and the editing level fold change >2 are considered to reach statistical significance in differential editing and considered ADAR3 M3 editing sites. Annotations for overall A-to-I sites were done with HOMER (http://homer.ucsd.edu/homer/ngs/). Sequence motif for highest confidence ADAR3 M3 editing sites were generated using WebLogo 3.

### Confirmation of editing by Sanger sequencing

Nested RT-PCR was performed on total RNA samples isolated from U87 cells transfected with hADAR3 M3 or hADAR3 M3 E434A plasmid DNA as described above for RNA-seq using Access RT-PCR kit (Promega) for 15 cycles and then followed by Phusion Hot Start DNA Polymerase (ThermoScientific) for the second PCR of 25 cycles. The resulting PCR product was purified by agarose gel and Qiagen Gel Extraction kit. The product was subjected to Sanger sequencing and sequence traces were analyzed by 4Peaks (Nucleobytes) to quantify percent editing.

### ADAR3/RNA immunoprecipitation assay and quantification by RT-qPCR

Human glioblastoma cell line U87 was grown and maintained in DMEM (Gibco) supplemented with 10% FBS (Invitrogen, Carlsbad, CA, USA), 100 units/ml penicillin and 100 μg/ml streptomycin in 5% CO_2_ at 37°C. Once cultivated cells reached 70–90% confluency, 1.5 × 10^5^ cells were seeded into 24-well plate. Cells were transfected 24 h later with 750 ng of hADAR3 wild type plasmid using Lipofectamine 2000 (ThermoFisher Scientific). After 48 h, cells were lysed with 150 μl of IP Lysis Buffer (Pierce) supplemented with Halt Protease Inhibitor (ThermoFisher Scientific) and RNase Inhibitor (NEB) for 20 min at 4°C with gentle agitation. Cell lysates were then cleared by centrifugation at 13 000 rpm for 15 min and 50 μl of lysate was incubated with anti-HA magnetic beads (Pierce) for 4 h at 4°C with gentle agitation. Magnetic beads and HA-tagged hADAR3 binding complex was washed three times with IP Lysis Buffer (Pierce) supplemented with Halt Protease Inhibitor (ThermoFisher Scientific) and RNase Inhibitor (NEB). Samples were then boiled in 0.1% SDS for 5 min.

Lysate prior to immunoprecipitation (input) and after immunoprecipitation (IP) were treated with RQ1 RNase-free DNase (Promega). RNA was then isolated using Trizol Reagent (ThermoFisher Scientific) and precipitated. RNA was resuspended in nuclease-free water and used for RT-qPCR. For RT-qPCR, RNA was diluted for a 20 μl reaction using TaqMan™ RNA-to-C_T_™ 1-Step Kit (ThermoFisher Scientific). TaqMan gene expression probes (DUSP1, EGR1, GAPDH, β-actin and 18S rRNA) (ThermoFisher Scientific) were used. Samples were amplified using standard qPCR program for TaqMan™ RNA-to-C_T_™ 1-Step Kit and is described as follows: initial holding at 48°C 15 min, followed by denaturation at 95°C 10 min, and further followed by 40 cycles of: 95°C 15 s and 60°C 1 min. The threshold cycle (C_t_) was set using the software Bio-Rad CFX Maestro (BioRad) on CFX Connect™ Real-Time System (BioRad). Transcript fold enrichment was determined using 18S rRNA as reference gene and by comparing input and post immunoprecipitation samples using the following equation:

$$\begin{eqnarray*}&&{\rm{Transcript}}\,{\rm{Fold}}\,{\rm{Increase}} = \\ && {2^{ - \left( {({\rm{Gene}}\,{\rm{of}}\,{\rm{Interes}}{{\rm{t}}_{{\rm{ADAR}}3\,{\rm{HA}}\,{\rm{tag}}\,{\rm{IP}}}} - {\rm{Gene}}\,{\rm{of}}\,{\rm{Interes}}{{\rm{t}}_{{\rm{input}}}}}) - ( {18{\rm{S}}\,{\rm{rRN}}{{\rm{A}}_{{\rm{ADAR}}3\,{\rm{HA}}\,{\rm{tag}}\,{\rm{IP\ }}}} - 18{\rm{S}}\,{\rm{rRN}}{{\rm{A}}_{{\rm{input}}}}} )\right)}}\end{eqnarray*}$$

### Transcript abundance by RT-qPCR Nested-RT PCR

Human glioblastoma cell line U87 maintenance and ADAR3 plasmids transfection are as described above. After 48 h of transfection, cells were lysed and total RNA was collected using RNAqueous 4PCR kit (Life Technologies) and DNase treated with RQ1 RNase-free DNase (Promega). For RT-qPCR, total RNA was used from cells overexpressing ADAR3 wild type or control (human ADAR2 deaminase domain mutant, ADAR2-D E396A). TaqMan gene expression probes (DUSP1, EGR1, GAPDH, β-actin and 18S rRNA) (ThermoFisher Scientific) were used. RT-qPCR was done with the TaqMan™ RNA-to-C_T_™ 1-Step kit following protocols as described above. Transcript fold increase was determined using 18S rRNA as reference gene and compared to empty pcDNA3.1 plasmid transfection samples using the following equation:

$$\begin{eqnarray*}&&{\rm{Transcript}}\,{\rm{Fold}}\,{\rm{Increase}} = \\ &&\, {2^{ - \left( {({\rm{Gene}}\,{\rm{of}}\,{\rm{Interes}}{{\rm{t}}_{{\rm{ADAR}}3}} - {\rm{Gene}}\,{\rm{of}}\,{\rm{Interes}}{{\rm{t}}_{{\rm{empty}}\,{\rm{pcDNA}}3.1}}}) - ( {18{\rm{S}}\,{\rm{rRN}}{{\rm{A}}_{{\rm{ADAR}}3}} - 18{\rm{S}}\,{\rm{rRN}}{{\rm{A}}_{{\rm{empty}}\,{\rm{pcDNA}}3.1}}}) \right)}}\end{eqnarray*}$$

## RESULTS

### Activating mutations in the ADAR3 deaminase domain predicted by Janus

**Figure 1.**
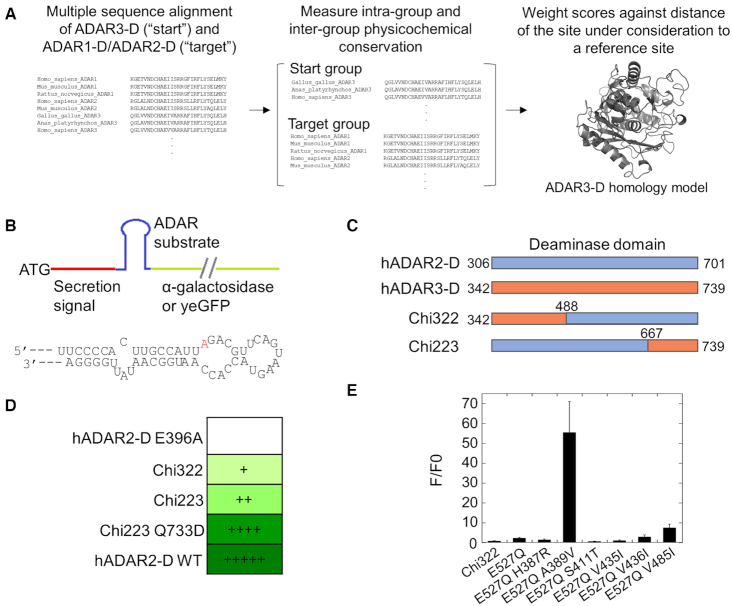
Janus prediction and hADAR2-D/hADAR3-D chimeras for testing the importance of mutations predicted by Janus. (**A**) Workflow of Janus program for predicting mutations important for ADAR3 activation. (**B**) Top: Scheme of the reporters for testing A-to-I editing activity (26,27). The colorimetric reporter and the fluorescent reporter use α-galactosidase and yeGFP (yeast enhanced green fluorescent protein) as the reporter protein, respectively. The secretion signal sequence exists only in the colorimetric reporter but not the fluorescent reporter. Bottom: sequence of the ADAR substrate used in the reporters. The editing site is colored in red. (**C**) Scheme of composition of hADAR2-D/hADAR3-D chimeras. The numberings in the chimeras are based on hADAR3 sequence. (**D**) A-to-I editing activity of the chimeras evaluated with the colorimetric reporter assay. ‘+’ indicates that yeast colonies started turning green after >30 days of incubation; ‘++’ indicates 9 days; ‘++++’ indicates 4 days; ‘+++++’ indicates 2 days. (**E**) The effect of mutations predicted by Janus on Chi322 activity using the fluorescent reporter assay. Mutations were introduced into Chi322. F/F0 is the ratio of fluorescence of cells expressing each mutant construct divided by fluorescence of cells expressing hADAR2-D inactive mutant E396A. Error bar indicates SD, *n* ≥ 3.

We sought first to convert ADAR3 into an active deaminase with limited mutations. In the absence of detailed structural information for wild type ADAR3, the bioinformatics program Janus was used to predict mutations required to revive activity. Janus is a bioinformatics tool that predicts and ranks mutations that can allow for functional interconversion of two structurally similar, but functionally distinct proteins. The program uses multiple sequence alignment information from the two proteins and scores the differences in conservation and covariation of physicochemical properties of each residue at a given position of the alignment. The score is then weighed by the inverse distance of the residue to a given reference site from a structure, usually the active site. A ranked list of predicted mutations in order of importance is generated in the end (21,22). We focused the application of Janus on the isolated deaminase domain of ADAR3 (ADAR3-D). Predictions employed ADAR3-D sequences from different organisms as the ‘start’ group that lacks activity and combined the sequences of both ADAR1 and ADAR2 deaminase domains (ADAR1-D and ADAR2-D) as the ‘target’ group that has activity (Figure 1A). Both ADAR1-D and ADAR2-D were used as the target group to achieve maximum stringency in defining residues critical to catalytic activity. Janus mutation scores were weighted by the distance of the residue to a reference site, which was the side chain OE2 atom of E434 in the ADAR3 homology model, the presumed proton transfer residue in the active site of ADAR3 (Figure 1A) (8,10). The 11 highest ranking Janus-predicted mutations are listed in Table 1.

**Table 1.** Top mutations for activating ADAR3 predicted by Janus using E434^a^ in active site as reference site

Score^b^	Predicted change	Score	Predicted change
100	V485I	48	H496F
72	A389V	43	V435I
71	T556S	42	V436I
63	V586L	38	S411T
59	V598A	23	Q549R
48	H387R		

^a^Numbering is based on human ADAR3.

^b^Normalized scores with highest scoring mutation set to 100.

### Converting ADAR3 into an active deaminase

A fluorescent reporter assay and a colorimetric reporter assay previously described were used here to evaluate the importance of Janus-predicted mutations to activation of ADAR3 (26,27) (Figure 1B). The assays link the deamination of adenosine in a 5′-UAG-3′ stop codon to the expression of either yeast enhanced green fluorescent protein, which fluoresces inside yeast cells, or α-galactosidase, which is secreted from yeast cells and turn yeast colonies green upon reaction of the x-α-Gal substrate present on the culture plate (Figure 1B). These two assays are advantageous at providing quantitative evaluation of A-to-I editing activities and reporting on low editing activities, respectively.

We focused on the top 11 mutations predicted by Janus listed in Table 1. Initial efforts included constructing an ADAR3 mutant library comprising mutants introduced with all possible combinations of these mutations. The resulting library was estimated to cover >100-fold of the theoretical diversity of the designed library (2^11^ = 2048), ensuring examination of every unique library member. The library was screened with fluorescence-activated cell sorting using the fluorescent reporter assay. No cells with outstanding fluorescence intensities were identified; nevertheless top 2% most fluorescent cells of the whole population screened (∼1 million) were collected and ADAR3 libraries plasmids regenerated from the harvested cells and transformed to yeast cells harboring the colorimetric reporter (Supplementary Figure S1). The resulting transformants were plated on the colorimetric plates and >2000 colonies were incubated at 30°C for an extended period (>30 days) for a green phenotype. However, these two layers of screening failed to identify any positive hit from this library, indicating that the top eleven mutations do not cover all necessary mutations needed to activate ADAR3.

We next assessed the importance of each of the top 11 mutations for activating ADAR3. This involved generating chimeric ADAR2/ADAR3 proteins. ADAR2-D and ADAR3-D share high sequence similarity and are therefore likely to generate stable chimeras. A series of hADAR2-D/hADAR3-D chimeric proteins were generated, with each comprising ∼1/3 of hADAR3-D sequence and 2/3 of hADAR2-D sequence (Figure 1C and Supplementary Figure S2A). The junctions between sequences from different origins were chosen at the homologous sites in the unstructured loops (according to the ADAR2-D crystal structure) to avoid perturbing local secondary structures (Supplementary Figure S4) (10). The chimeric proteins were tested using the colorimetric screening assay and two chimeras (Chi322 and Chi223) appeared to be active (Figure 1C, D, Supplementary Figure S2).

Among the top eleven mutations, six (H387R, A389V, S411T, V435I, V436I, V485I) are located in the ADAR3 sequence in Chi322. We set out to evaluate the effect of each individual mutation on the activity of Chi322 using both the fluorescent reporter assay and the colorimetric reporter assay. We found that the activity of Chi322 was too low to generate above-background signal in the fluorescent assay. Thus, to generate quantifiable fluorescence signal, an E to Q mutation at the base flipping residue (E527Q in hADAR3) known to enhance ADAR editing activity was introduced in Chi322 (26,37). The resulting mutant generated a fluorescence signal of ∼2.3-fold of that of the negative control (Figure 1E). On the basis of Chi322 E527Q, each mutation was further introduced individually, and activities of the resulting mutants were evaluated using the fluorescent reporter assay (Figure 1E). The fold change of fluorescence signals relative to Chi322 E527Q was calculated for a quantitative evaluation of the effect of the introduced mutation on the editing activity. Importantly, we found that mutation A389V increased the fluorescence signal by more than 20-fold, suggesting a substantial increase in the activity of the chimeric protein. Another mutation, V485I, increased the fluorescence intensity by ∼3-fold, suggesting that this mutation is also beneficial for activating the ADAR3 deaminase, albeit to a lesser extent. The other mutations did not substantially increase the fluorescence signal, suggesting that they do not contribute to ADAR3 deaminase activity. Consistent with results from the fluorescent reporter assay, the colorimetric reporter assay showed that A389V mutation had the largest effect on editing activity of Chi322 and V485I had a beneficial but lesser effect whereas other mutations tested showed no effect (Supplementary Figure S3A–C).

The other active chimeric protein that was identified, Chi223, contains hADAR3 sequence spanning aa667- aa739. There is no mutation predicted by Janus for the ADAR3 region in Chi223; however, the activity of Chi223 is not optimal suggesting activity-inhibitive elements in this protein segment. The lack of Janus-predicted mutations in this region is likely due to the large distance of this region from the reference site chosen in the original prediction protocol, and the 1/distance mutation weighting scheme employed here. The ADAR3 segment in Chi223 contains a region bound to an inositol hexakisphosphate (IHP) molecule. The IHP molecule in ADAR is deeply buried in the protein and is crucial for both the structure and activity of ADARs (38).

We carried out a second Janus prediction using NZ of K700 near the IHP molecule as the reference site, generating a new list of candidate mutations shown in Table 2. We tested the top mutation, Q733D, in the background of Chi223. Importantly, introducing this mutation to Chi223 clearly increased signal in the colorimetric reporter assay to a level comparable to that of wild type ADAR2 (Figure 1D, Supplementary Figure S3D), indicating this mutation is beneficial for activating ADAR3.

**Table 2.** Top mutations for activating ADAR3 predicted by Janus using K700^a^ at IHP site as reference site

Score^b^	Predicted change	Score	Predicted change
100	Q733D	77	L442I
93	V709A	77	V436I
86	H496F	76	T556S
83	V598A	74	Q549R
78	H443R	68	H387R

^a^Numbering is based on human ADAR3.

^b^Normalized scores with highest scoring mutation set to 100.

**Figure 2.**
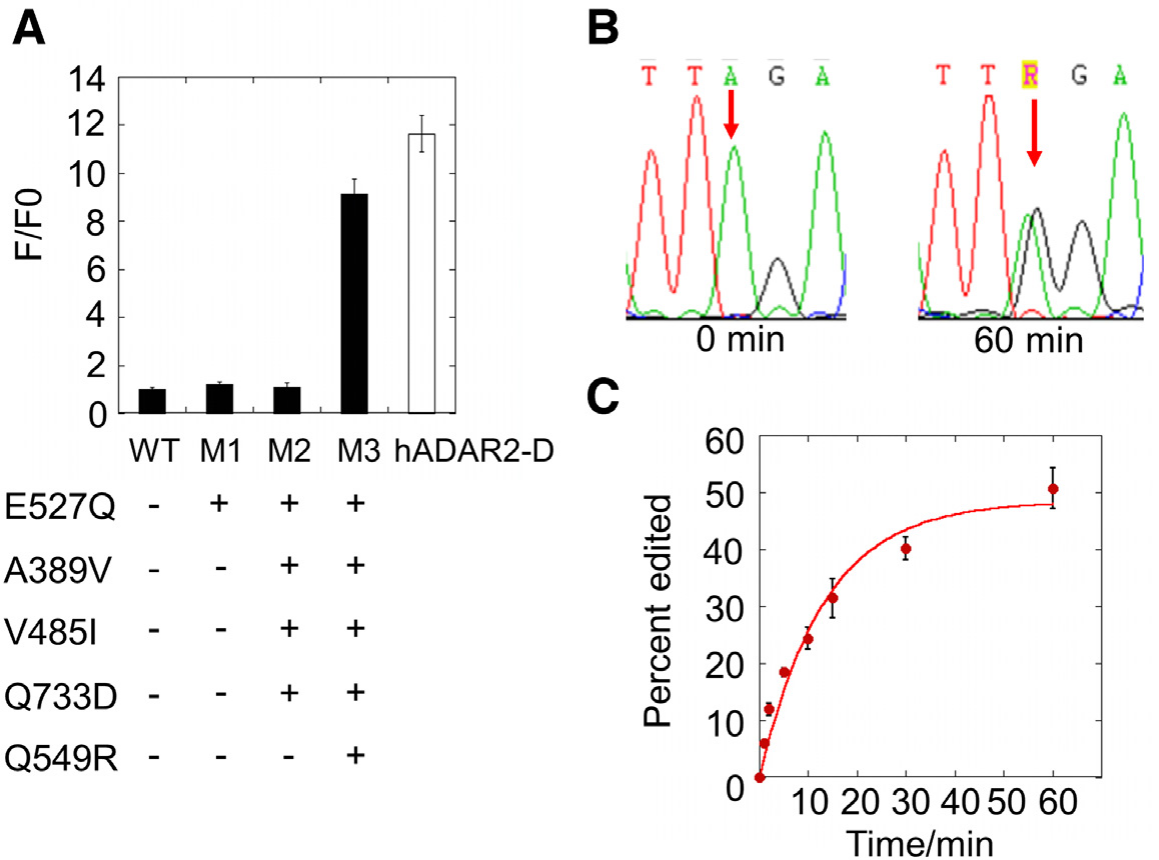
Converting human ADAR3 into an active deaminase by introducing five mutations. (**A**) Evaluation of RNA editing activity of hADAR3-D mutants using the fluorescent reporter assay. WT is the wild type hADAR3-D. M1, M2 and M3 are hADAR3-D mutants introduced with mutations as shown. F/F0 is the ratio of sample fluorescence divided by negative control (inactive mutant) fluorescence. Error bar indicates SD, *n* ≥ 3. (**B**) Editing of the catalytically active ADAR3 mutant M3 on BDF200 RNA substrate at t = 0 min and t = 60 min. The editing site is shown by an arrow. (**C**) Fraction edited by ADAR3-D M3 in the BDF2 model substrate RNA as a function of time. Error bar means SD. *n* ≥ 3.

It is known that an E to Q mutation at the base flipping residue of either ADAR1 or ADAR2 increases activities of both enzymes substantially (26,37). We converted the putative base flipping residue of human ADAR3 (E527) to a Q, giving rise to a single point mutant hADAR3-D protein (M1) with no detectable editing activity, indicating that the E527Q mutation alone is not sufficient to activate the deaminase (Figure 2A). We then introduced the three activity-improving mutations identified above (A389V, V485I and Q733D), giving rise to mutant hADAR3-D M2. With the fluorescent reporter assay, hADAR3-D M2 generated no positive signal (Figure 2A), indicating additional activity-repressing elements in ADAR3. Previous studies showed that residue R510 in ADAR2, which contacts the 3′ phosphodiester of the base opposite the editing site in the RNA (i.e. the orphan base), when mutated to the corresponding residue in ADAR3 (glutamine at position 549), led to a substantial decrease in the deamination efficiency of ADAR2 (10). This suggests that Q549 in ADAR3 may be activity inhibiting. Importantly, Q549R is one of the top ranked mutations predicted by Janus for activating ADAR3 using either reference site (Table 1, Table 2). We thus further introduced the Q549R mutation into hADAR3-D M2, giving rise to mutant hADAR3-D M3. hADAR3-D M3 generated a clear fluorescence signal whose intensity was comparable to that generated by wild type hADAR2-D (Figure 2A and Supplementary Figure S4)).

hADAR3-D M3 was overexpressed, purified and tested for its editing activity *in vitro* on model RNA substrates. The mutant protein showed clear editing activity on a segment of the yeast bromodomain factor 2 mRNA, the same type of substrate used in the reporter assays (Figure 1B), with a *k*_obs_ of 0.076 ± 0.0045 min^−1^ (Figure 2B, C) (26). In contrast, under the same condition, the wild type ADAR3-D showed no editing. hADAR3-D M3 also edits a site from the human glioma factor 1 (GLI1) mRNA (Supplementary Figure S5), further demonstrating the deaminase activity of this enzyme *in vitro*.

Next, we tested whether hADAR3-D M3 could deaminate previously identified editing targets of hADAR1-D and hADAR2-D from the *S. cerevisiae* transcriptome. Here we tested the editing activity of hADAR3-D M3 on these RNAs by overexpressing hADAR3-D M3 in yeast. With hADAR3-D M3 overexpressed in yeast, we observed efficient editing on nine known substrates (Supplementary Table S1 and Supplementary Figure S6). Interestingly, although the hADAR3-D M3 sequence is highly similar to that of hADAR2-D, their selectivity among these nine targets is clearly different (Supplementary Figure S6A). We speculate that this is due to the substantial differences in their 5′ binding loops (Supplementary Figure S6B) (10,39). On these RNAs, we further tested whether the full length hADAR3 bearing the M3 mutations could also perform editing in yeast cells. We overexpressed the full-length hADAR3 M3 in yeast and found a clear, but generally lower editing compared to hADAR3-D M3 (Supplementary Figure S6A). Western blots showed that the full-length hADAR3 M3 was expressed to a much lower level than the hADAR3-D M3 (Supplementary Figure S6C), indicating that the low editing levels introduced by full-length hADAR3 M3 are likely a result of its low expression level in the yeast cells.

Mutations that activate ADAR3 appear to be involved in stabilizing the base-flipped conformation necessary for catalysis (see Discussion). Indeed, using the homology model of hADAR3-D, we performed molecular dynamics simulations comparing RNA complexes of hADAR2-D, hADAR3-D and hADAR3-D M3. These simulations suggest that the distance between the zinc-coordinated hydroxyl group and the C6 position of the reactive purine in the complex with wild type ADAR3-D is longer than those in complexes of ADAR2-D and ADAR3-D M3 (Supplementary Figure S7).

### RNA-seq to identify endogenous binding targets of hADAR3

Having shown that the mutant hADAR3-D M3 could efficiently catalyze deamination on various RNAs both *in vitro* and in yeast cells, we turned our attention to identifying RNA substrates in human cells. McMahon *et al.* developed the TRIBE method in which RNA binding proteins (RBPs) are fused to a deaminase domain of an ADAR to detect the binding targets of the RBPs (40). The deaminase domain of ADAR causes A-to-I editing thus placing inosine marks on the bound RNA, which are then identified by RNA-seq. The method is advantageous over the conventional CLIP-seq method in that it is more specific, requires less material, and is more efficient for double stranded RNA binders (41). Inspired by this method, we envisioned a similar approach that would use full length hADAR3 M3 bearing the five mutations to identify the binding targets of wild type human ADAR3.

**Figure 3.**
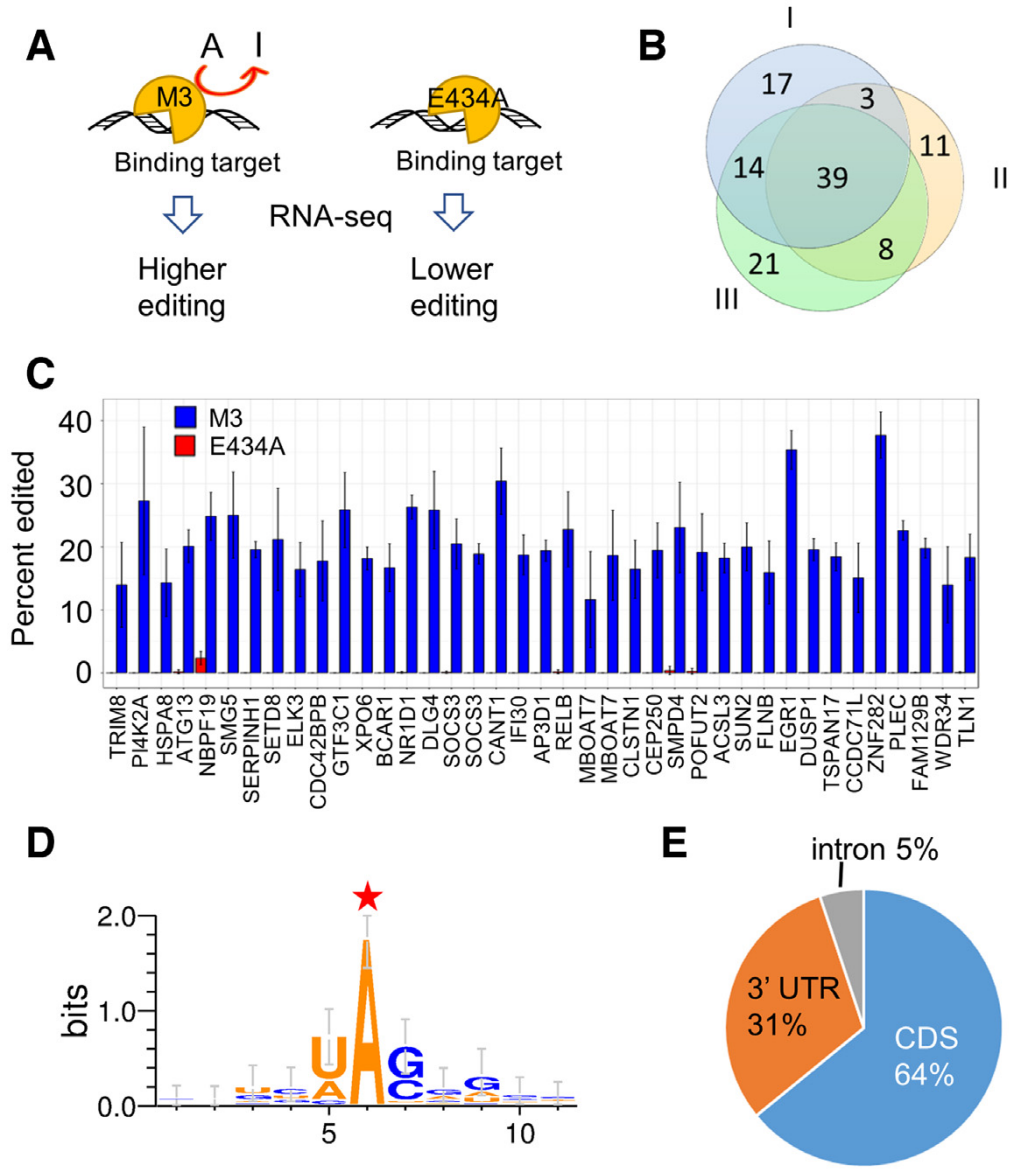
ADAR3-seq to identify cellular binding targets of hADAR3. (**A**) Schematic of ADAR3-seq. (**B**) Overlap of candidate editing sites identified from biological replicate I, II and III. (**C**) Editing levels of the 39 candidate editing sites in the most confident group upon hADAR3 M3 overexpression versus inactive E434A overexpression calculated from data of RNA-seq. X-axis is the names of the genes where the candidate editing sites are located. Statistics of reads counts for these 39 sites across all samples show that the lowest read count is 47, highest is 2673 and average is 333. Thus, read count is not a limitation for editing level quantification. (**D**) Sequence motif surrounding the candidate editing site (labeled with a red star) in the most confident group. (**E**) Pie chart depicting the region distribution of the candidate editing sites in the most confident group.

RNA binding selectivity by ADARs is controlled by the double stranded RNA binding domains and the RNA binding surfaces in the deaminase domains (10,42,43). Importantly, none of the five mutations introduced in M3 is on a primary RNA recognition surface of the deaminase domain (i.e. 5′ or 3′ binding loop), although two mutations (E527Q and Q549R) are expected to enhance contacts important for stabilizing the base-flipped RNA conformation necessary for deamination (see below) (10,44). Therefore, RNA binding selectivity by hADAR3 is expected to be minimally altered by the mutations introduced, and the editing targets of the active full length hADAR3 M3 mutant should represent binding targets of the wild type hADAR3. As a control, an inactivated mutant of hADAR3 M3 generated by mutating the proton transfer residue E434 to an alanine was used to account for competitive binding by hADAR3. Overexpressing the full-length hADAR3 M3 in human cells should cause elevated editing on bound RNA substrates compared to the control E434A mutant, which could be identified by RNA-seq (Figure 3A).

Wild type hADAR3 is normally only expressed in brain, therefore we chose a brain-derived tumor cell line, glioblastoma U87 MG, to identify biologically relevant hADAR3 binding targets. The full-length hADAR3 M3 and its inactive mutant E434A were similarly expressed in U87 cells (Supplementary Figure S8).

Before identifying potential transcriptome-wide editing targets of hADAR3 M3, we performed reverse transcription followed by PCR to amplify regions surrounding the editing site in glioma factor 1 (GLI1) mRNA on total RNA isolated from cells to evaluate the method. The GLI1 mRNA is a substrate of both hADAR1 and hADAR2 (45) (and was shown to be efficiently edited by hADAR3-D M3 *in vitro* (Supplementary Figure S5)). We found that GLI1 editing was elevated in cells transfected with the full length human hADAR3 M3, compared to cells transfected with the E434A (Supplementary Figure S8B, C). This result validates the method and suggests that the GLI1 mRNA is a binding target of hADAR3.

RNA-seq was used to identify transcriptome-wide binding targets of hADAR3. Each replicate consists of an hADAR3 M3 experimental sample and an E434A control sample. Overall more than 20,000 A-to-I editing sites were identified for each replicate, among which 42–46% are overlapped with RADAR, a comprehensive repository of known A-to-I sites (Supplementary Figure S9) (46). Each replicate also covers around 17- 21% of editing sites reported by Bahn *et al.* for U87 MG cell line (Supplementary Figure S9) (47); the low overlap is likely because we and Bahn *et al.* have each identified a subset of editing sites in this cell line. Approximately half the population of the A-to-I editing sites we identified are located in repetitive regions (Supplementary Figure S9A), in agreement with previous knowledge that most A-to-I editing events occur in the repetitive regions. To identify ADAR3 M3 editing sites, among the overall editing sites we further looked for sites whose editing levels are elevated in the active ADAR3 expressed sample compared to the inactive mutant expressed sample, following the bioinformatics criteria we applied (see methods). Three biological replicates identified 73, 61 and 82 elevated editing sites (relative to the control, adjusted *P* < 0.05) respectively which exhibited high overlap, indicating the reproducibility of the method and reliability of the identified sites (Figure 3B, Supplementary Table S2). Sixty-four sites are identified in at least two replicates and are considered more confident than other sites, while 39 sites show up in all three replicates and are considered the most confident sites (Supplementary Tables S3). We focused on the highest confidence sites for further analysis. Figure 3C shows statistics from the three replicates confirming that the editing levels at the highest confidence sites are increased by overexpression of hADAR3 M3 compared to overexpression of the inactive mutant.

**Figure 4.**
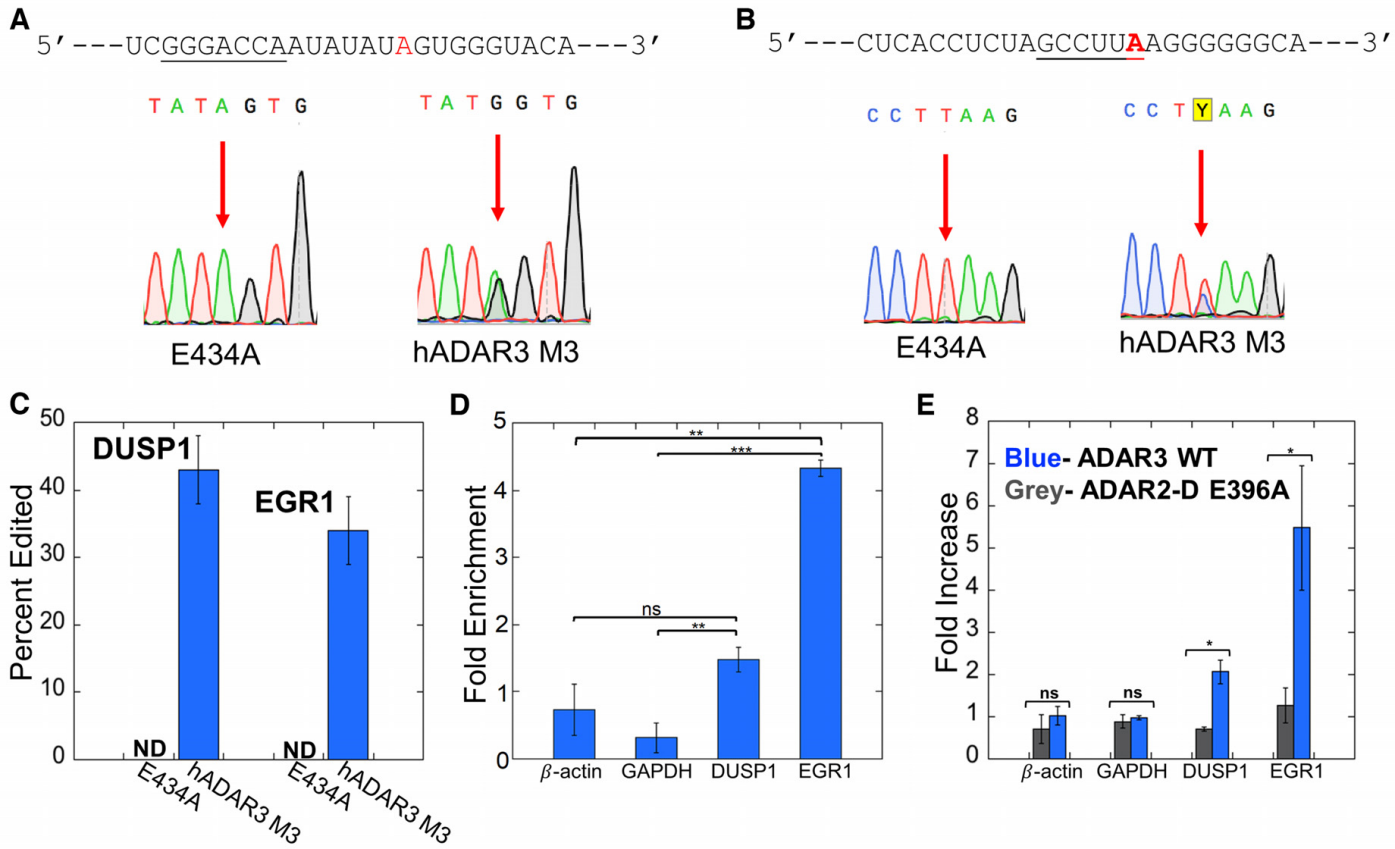
ADAR3 target sites in 3′ UTRs of DUSP1 and EGR1. (**A**) Editing site for ADAR3 M3 in DUSP1 3′ UTR. (Top) Sequence adjacent to DUSP1 editing site with deamination site highlighted red and putative miR133a binding site underlined. (Bottom) Confirmation of editing by hADAR3 M3 at this site in U87 cells by Sanger sequencing. (**B**) Editing site for ADAR3 M3 in EGR1 3′ UTR. (Top) Sequence adjacent to EGR1 editing site with deamination site highlighted red and putative miR506 binding site underlined. (Bottom) Confirmation of editing by hADAR3 M3 at this site in U87 cells by Sanger sequencing. (**C**) Quantification of editing by hADAR3 M3 at the DUSP1 and EGR1 3′ UTR editing sites U87 cells. Plotted values are averages of three independent replicates ± standard deviation. (**D**) Fold enrichment of RNA transcripts in a RIP-qPCR experiment showing interaction of HA-tagged wild type ADAR3 with DUSP1 and EGR1 RNAs in U87 cells. The fold enrichment was calculated by comparing RNA levels in samples after and prior to HA-ADAR3 immunoprecipitation with the 18S rRNA as the internal reference gene. Plotted values are averages of three independent replicates ± standard deviation. Statistical differences between groups were determined by *t* tests. **P < 0.01; ***P < 0.001; ns, not significant. (**E**) Effect of expression of wild type ADAR3 in U87 cells on transcript levels measured by RT-qPCR (blue bars). The RNA fold increase was calculated relative to samples transfected with empty vector with 18S rRNA as internal reference gene. Expression of an inactive mutant of the human ADAR2 (ADAR2-D E396A) does not alter levels of these transcripts (grey bars). Plotted values are averages of three independent replicates ± standard deviation. Statistical differences between groups were determined by *t* tests. *P < 0.05; **P < 0.01; ***P < 0.001; ns, not significant.

The sequence motif surrounding the highest confidence sites showed a preference for U and A 5′ to the editing site and G and C 3′ to the editing site (Figure 3D), in agreement with previously observed nearest-neighbor preferences for ADARs, suggesting these are indeed hADAR3 M3 editing sites. The editing sites are mainly found in coding regions (64%), with the rest distributed in 3′ UTR (31%) and intron (5%) (Figure 3E). These RNAs encode proteins with a variety of functions, such as transcription regulation (EGR1, GTF3C1, NR1D1, ELK3 and SETD8), cell signaling (DUSP1), mRNA decay (SMG5), inflammation (SOCS3), cell autophagy (ATG13) and neuronal signaling (DLG4). We searched two different online databases of known RNA editing sites (REDIportal (2017) and RADAR (2014)) and a recently reported compilation of known human editing sites (46,48,49) and found that many of the 39 highest confidence ADAR3 target transcripts are known substrates for either ADAR1 or ADAR2. However, hADAR3 M3 edits different adenosines in each of these transcripts compared to ADAR1 or ADAR2, except for PI4K2A, SMPD4 and IFI30. In addition, two of the highest confidence ADAR3 target transcripts identified here (DUSP1 and NBPF19) were not reported to be edited by ADAR1 or ADAR2 in any tissue. We validated a few hADAR3 M3 editing sites located in 3′ UTR (DUSP1 and EGR1) and CDS (NR1D1, PI4K2A and SMG5) using reverse transcription followed by PCR and Sanger sequencing (Figure 4 and Supplementary Figure S10).

### ADAR3 binds to transcripts for immediate early genes DUSP1 and EGR1 and regulates their expression levels in cells

Among the novel ADAR3 binding genes identified, we focused our attention on two particularly interesting transcripts; mRNAs of DUSP1 and EGR1. DUSP1 mRNA encodes for dual specificity phosphatase 1, also referred to as MKP-1 (MAP kinase phosphatase 1). This enzyme dephosphorylates and inactivates MAP kinases such as ERK, JNK and p38 and is an important negative feedback regulator of MAP kinase activation (50–52). Another hADAR3 M3 editing target of note is EGR1 (early growth response 1, a.k.a. Zif268) (53,54). EGR1 is a transcription factor implicated in neuronal plasticity and memory formation (53,54). Both EGR1 and DUSP1 are activity-dependent immediate early genes (IEGs) that respond to stimuli in the brain (53,55–57), indicating that ADAR3 may be involved in cognitive processes through interaction with these genes. We validated editing by hADAR3 M3 on the DUSP1 and EGR1 transcripts in U87 cells using reverse transcription followed by PCR and Sanger sequencing. In both cases, hADAR3 M3 efficiently deaminates a site in the transcript's 3′ UTR (Supplementary Table S1, Figure 4A–C), suggesting that wild type hADAR3 binds to each RNA at the 3′ UTR. To confirm binding of wild type hADAR3 to DUSP1 and EGR1 transcripts, we expressed HA tagged hADAR3 in U87 cells and immunoprecipitated the tagged protein followed by determination of the fold enrichment of DUSP1 and EGR1 transcripts by the protein via RT-qPCR. Our data showed that compared to samples prior to immunoprecipitation, DUSP1 and EGR1 transcripts were enriched by around 1.5-fold and 4.5-fold respectively, as opposed to the control genes β-actin and GAPDH which were not enriched, demonstrating the interaction between hADAR3 and DUSP1 and EGR1 transcripts (Figure 4D). Moreover, the adenosine deaminated further localizes the ADAR3 binding site to a specific position within the 3′ UTR. In each case this corresponds to a location proximal to a predicted binding site for a miRNA (miR133a for DUSP1 and miR506 for EGR1) (Figure 4A, B). These observations suggested that ADAR3 binding may have an effect on expression levels of DUSP1 and EGR1 by competing with miRNAs or other RNA-binding proteins that control transcript stability through 3′ UTR interactions. To test this idea, we expressed wild type hADAR3 in U87 cells and used RT-qPCR to assess transcript levels for both DUSP1 and EGR1. Importantly, hADAR3 expression caused an increase in measured transcript levels for both DUSP1 (2.1 ± 0.3-fold) and EGR1 (5 ± 1-fold), whereas β-actin and GAPDH transcripts are unaffected (Figure 4E). For comparison, we also expressed an RNA-binding and deaminase deficient mutant of human ADAR2 (ADAR2-D E396A) and this had no effect on DUSP1 or EGR1 transcript levels (Figure 4E).

## DISCUSSION

A significant knowledge gap in our understanding of ADAR3 function is its RNA binding profile. Traditionally, ADAR1 and ADAR2 substrates are identified based on the A-to-I editome, but this is not possible for ADAR3 due to its lack of RNA editing activity. Thus, alternative strategies, such as CLIP-seq, are required to identify binding substrates for ADAR3. However, CLIP-seq requires large amounts of RNA as starting materials, suffers from high level of non-specific crosslinking, exhibits low cross-linking efficiency for dsRNA binding proteins as well as prevents downstream analysis of binding sites due to the introduction of sequencing errors at the sites of crosslinking. We envisioned that an activated version of ADAR3 would facilitate the identification of RNA substrates for ADAR3 by putting inosine marks on substrates to which it binds. To this end, we engineered a catalytically active ADAR3 variant using a combination of computational prediction and functional screening. Five mutations (out of 739 amino acids; a mutation rate of 0.6%) engender RNA deaminase in human ADAR3. These efforts have provided insights into the catalytic incapability of the wild type protein (see below), as well as enabled us to use the pentamutant enzyme as a tool to identify potential cellular binding targets for ADAR3. Further studies into the novel ADAR3 substrates identified in this manner reveal that the wild type ADAR3 protein can regulate transcript levels for immediate early genes DUSP1 and EGR1, suggesting a novel role ADAR3 may play in mammalian nervous system (see below).

Earlier domain swapping experiments established that the N-terminal RNA binding region of human ADAR3 is functional, capable of binding both duplex and single stranded RNA (8,11). Yet no catalytic activity could be shown for the ADAR3 deaminase domain (8). This domain is highly similar to the deaminase domain found in human ADAR2 (50% identity and 70% similarity) and active site residues involved in zinc binding and proton transfers are present in the wild type ADAR3 sequence (1,8). However, residues outside an enzyme active site can also be important for catalysis, yet their identities and roles are more difficult to determine. To aid in identifying residues present in ADAR3 that may be responsible for its lack of deaminase activity, we turned to the Janus computer program, which predicts mutations required to interconvert structurally related yet functionally distinct enzymes. Using sequences for ADAR3 and the two active ADAR enzymes from different organisms as Janus input, residues conserved in the active ADARs but different in ADAR3s were identified and ranked based on physicochemical properties and distance from known functionally important reference points (e.g. the zinc-containing active site and IHP-binding site). Using this approach, we identified candidate mutations to convert ADAR3 into an active RNA deaminase. A subset of these mutations were then tested in ADAR2/ADAR3 chimeric proteins to determine their effect on activity.

**Figure 5.**
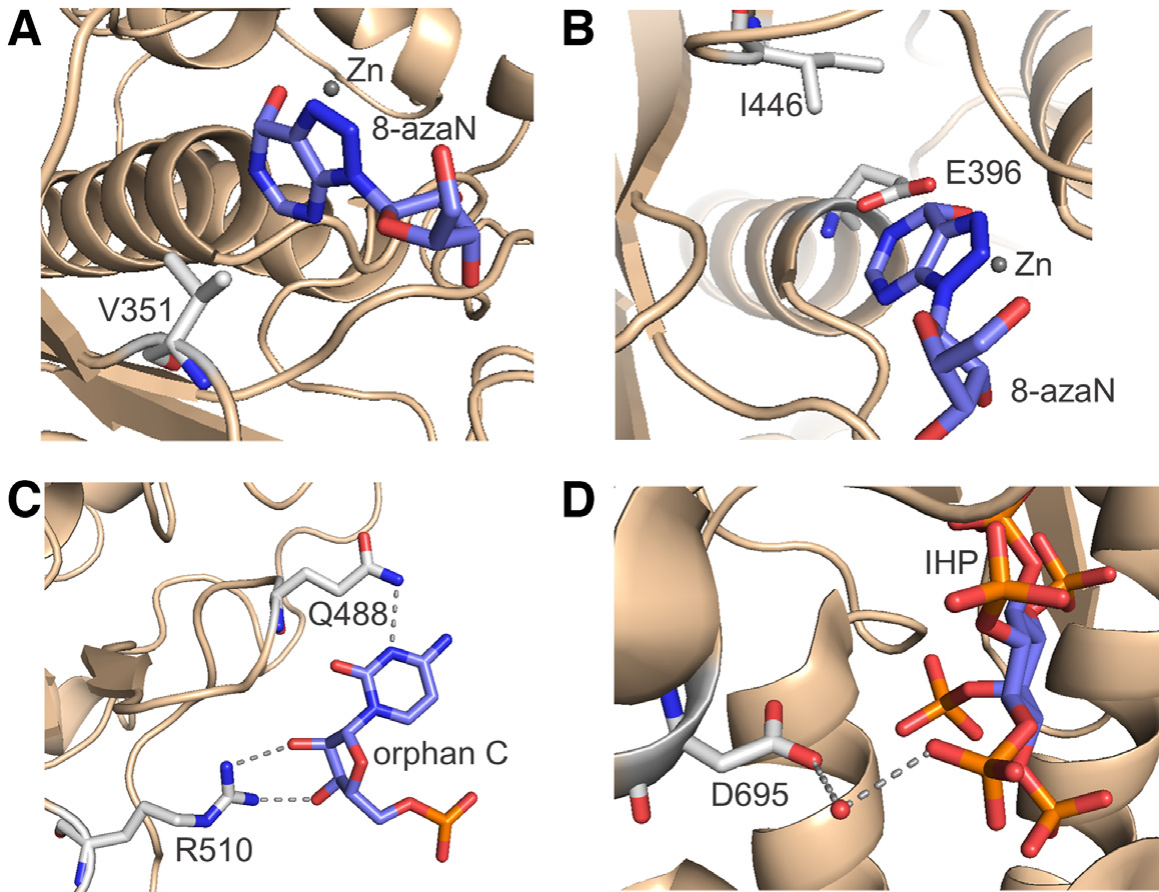
Interactions surrounding the hADAR3 mutations-corresponding locations in hADAR2 (10). (**A**) V351 (corresponding to A389 in hADAR3) mediates hydrophobic interactions with the edited base. (**B**) I446 (corresponding to V485 in hADAR3) facilitate anchoring the proton transfer residue E396. (**C**) Q488 and R510 (corresponding to E527 and Q549 respectively in hADAR3) contacts the orphan base of RNA substrate. (**D**) D695 (corresponding to Q733 in hADAR3) has a water-mediated contact to IHP.

With this strategy we identified four relatively conservative mutations (A389V, V485I, Q549R and Q733D) that, along with the known deaminase-activating mutation E527Q, activated the ADAR3 deaminase. These five amino acids are each involved in different aspects of ADAR catalysis (Figure 5).

The A389V mutation is at a location in ADAR3 corresponding to V351 in human ADAR2. In crystal structures of the ADAR2 deaminase domain bound to RNA bearing a transition state analog (8-azaN hydrate) at the editing site, the side chain of V351 provides a hydrophobic surface that anchors the edited base in position for catalysis (Figure 5A). An alanine at this position is likely to be less effective due to the shorter side chain. The V485I mutation corresponds to I446 in ADAR2. Here the side chain of the isoleucine residue contacts that of E396, positioning it for proton transfers to and from the reacting base during catalysis (Figure 5B). The Q549R and E527Q mutations alter residues involved in stabilizing the base-flipped conformation of the nucleotide opposite the editing site (i.e. the orphan base) during catalysis (Figure 5C). Finally, the Q733D mutation (corresponding to D695 in ADAR2) is located at the IHP binding site. The side chain of D695 is involved in water-mediated hydrogen bonding to an IHP phosphate, and a glutamine at this location is likely to disrupt this interaction (Figure 5D). Results from a molecular dynamics simulation were consistent with the notion that the five mutations stabilize a conformation of the protein-RNA complex involving close approach of the reactive zinc-hydroxide to adenine C6 (Supplementary Figure S5). Given the extent of changes necessary at residues involved in different aspects of adenosine deamination, we think it unlikely that wild type ADAR3 is a functional RNA deaminase.

Using the active human ADAR3 M3, we attempted to identify binding partners of the wild type protein with RNA-seq technology. In total, 103 confident positive hits were identified as hADAR3 M3 sites from the three biological replicates, with 39 appearing in all three replicates, 25 in two replicates and 49 in one replicate (Supplementary Table S2 and S3). Thus, 39 highest confidence hADAR3 M3 editing sites were identified here revealing not only the identity of transcripts that interact with ADAR3 but also the specific location of the binding site near the adenosine that is deaminated. The highest confidence hADAR3 M3 editing sites discovered here are mainly located in coding sequences (Figure 3E), This is likely due to that overexpression of hADAR3 M3 did not cause statistically significant changes in editing at most sites in the repetitive regions, thus they were not identified as hADAR3 M3 editing sites by our protocol. We believe that expression of ADAR3 along with knocking down ADAR1 and/or ADAR2 to decrease RNA editing background would significantly expand the ADAR3-editing list reported in this study. Besides, prior to performing RNA-seq, we found that editing on the Gli1 RNA, which is also edited by both ADAR1 and ADAR2, was improved from ∼10% upon overexpression of inactive hADAR3 M3 E434A to around 17% with overexpressed hADAR3 M3 (Supplementary Figure S8). However, this site was not identified by RNA-seq due to low read coverage of the gene. This suggests that additional positive sites might be identified with deeper sequencing. It is also possible that important ADAR3 binding RNAs could be missed by our approach if the RNA lacks an adenosine positioned to react in the binding site.

While this manuscript was in preparation, Mladenova *et al.* reported genes that are differentially expressed in response to ADAR3 deficiency (15). Importantly, among the ADAR3-dependent genes they reported, 14 overlap with positive hits identified here: BCAR1, DLG4, EGR1, FASN, CRKL, HK2, ZYX, MARK2, APBB1, TLE3, NFIX, KMT2B, PRKAR2A and ABL. These common targets identified in two independent studies suggest that they are true binding partners of ADAR3 and corroborate our method of using activated ADAR3 to identify binding partners of the wild type hADAR3. Indeed, we have confirmed via an immunoprecipitation followed by qPCR assay the binding of the wild type ADAR3 to two (DUSP1 and EGR1 transcripts) of the RNAs identified by our method (Figure 4D).

**Figure 6.**
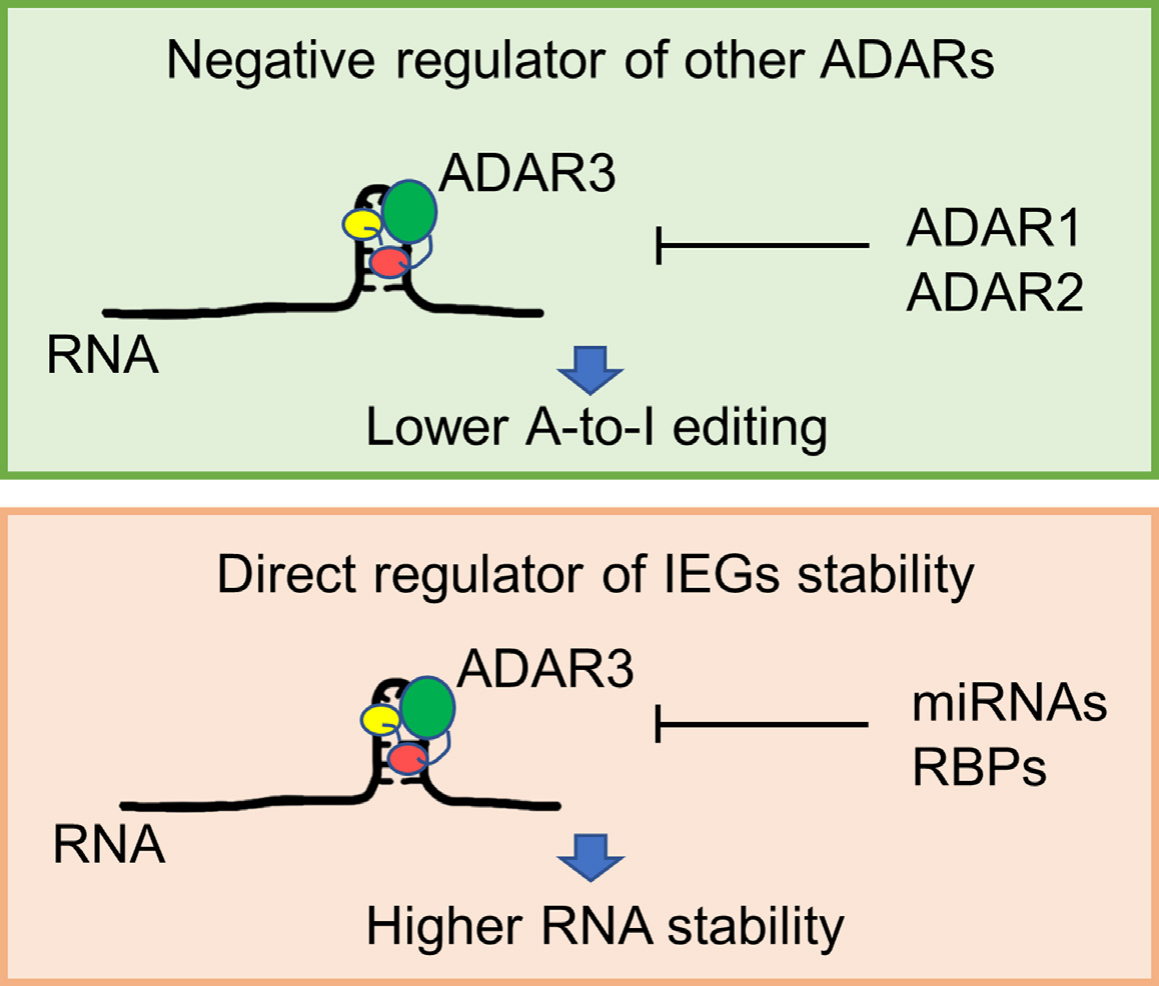
Known ADAR3 function as a competitor of ADAR1 and ADAR2 and proposed new function for ADAR3 as a direct regulator of IEGs RNA stability.

The importance of defining the RNA binding partners for ADAR3 is highlighted by recent discoveries that implicate ADAR3 in learning and memory and in various diseases, including acute lymphoblastic leukemia, hepatocellular carcinoma, brain tumors and Alzheimer's disease (58–61). Previously it was known that ADAR3 acts as a competitive binder for the other two ADARs thereby regulates RNA editing negatively (Figure 6). However other mechanisms by which ADAR3 might regulate RNA processing are unknown. Particularly intriguing among our findings were ADAR3 binding sites in 3′-UTRs for the immediate early genes (IEGs) EGR1 and DUSP1 (Figure 3, Figure 4). The expression of IEGs in response to new sensory experiences is important for changes to synapses, learning and memory (53,56,57). However, the precise mechanisms by which these expression levels are controlled are not completely understood (57). Interestingly, the ADAR3 M3 editing sites found in both the EGR1 and DUSP1 3′-UTRs are near predicted miRNA binding sites suggesting a possible competition between ADAR3 and miRNAs for the same sites (62). Furthermore, the stability and translation efficiency of DUSP1 mRNA are known to be regulated by protein binding to its 3′ UTR (63). Indeed, the dsRBD-containing protein NF90 binds the DUSP1 3′ UTR and increases transcript half life (63). The transcript stabilizing protein HuR also binds the DUSP1 3′-UTR (63). An earlier study demonstrated that HuR and ADAR1 cooperate to bind and stabilize specific mRNAs (64,65). Indeed, we found here that ADAR3 expression in U87 cells increases transcript abundance for both DUSP1 and EGR1. These results suggest that one mechanism by which ADAR3 could influence cognitive processes is by directly regulating transcript levels for certain IEGs through competitively binding to 3′ UTR which could be otherwise a target of miRNAs or other RNA-binding proteins (Figure 6). Additional experiments will be necessary to further test this hypothesis.

We also found that a large fraction of ADAR3 binding sites identified by our protocol are located in the CDS region of RNAs. It is possible that by binding to the CDS region of RNAs, ADAR3 could regulate RNA translation and/or splicing. Further studies will be necessary to determine the consequence of ADAR3 binding to these and other RNA targets identified here.

Looking forward, we envision that the active mutant ADAR3 M3 introduced in this study will be invaluable for future studies of ADAR3 structure and biology. Further improvement of the mutant will be possible, with potential application in site-directed RNA editing (66–68), given the efficient editing activity and distinct deamination specificity exhibited by the ADAR3 mutant compared to ADAR1 and ADAR2. Finally, our study exemplifies a success in combining a bioinformatics tool and functional screening to activate an intrinsically inactive protein. Similar approach may be used to activate catalytically inactive members in other protein families and utilizing the active versions of the proteins to study their function in the biological context may be widely appreciated by other fields.

## DATA AVAILABILITY

Data has been deposited to GEO under accession number GSE129142: https://www.ncbi.nlm.nih.gov/geo/query/acc.cgi?acc=GSE129142.

## Supplementary Material

gkz815_Supplemental_Files
