# Supplementary material for: RNA binding candidates for human ADAR3 from substrates of a gain of function mutant expressed in neuronal cells
Source: Nucleic Acids Res. 2019 Sep 25;47(20):10801–14. doi: 10.1093/nar/gkz815 (PMC6846710; doi:10.1093/nar/gkz815)
Supplement: gkz815_Supplemental_Files [file gkz815_supplemental_files.zip › SI ADAR3-gkz815-revision-Final-Correction.pdf]

Supporting Information

RNA Binding Candidates for Human ADAR3 from Substrates of a Gain of Function Mutant Expressed in Neuronal Cells

Yuru Wang, Dong hee Chung, Leanna R. Monteleone, Jie Li, Yao Chiang, Michael D. Toney and Peter A. Beal*

Department of Chemistry, University of California, One Shields Ave, Davis, CA 95616, USA.

*Corresponding author, tel: (530) 752-4132. E-mail: pabeal@ucdavis.edu

<u>Contents</u>

Figure S1 FACS to screen ADAR3 library covering top 11 mutations predicted by Janus....... S2

Figure S2 Colorimetric assay to evaluate activities of ADAR2-D/ADAR3-D chimeras............. S3

Figure S3 Colorimetric assay to evaluate effect of mutations predicted by Janus on the activities of Chi322 or Chi223 .... S4

Figure S4 Sequence alignment between three ADARs depicting chimera construction strategy and mutations introduced in hADAR3 M3 .... S5

Figure S5 hADAR3-D M3 editing on human GLI1 mRNA S6

Figure S6 Editing on yeast RNA substrates by hADAR3 M3 and sequence alignment of 5' binding loops between ADAR2 and ADAR3 .... S7

Figure S7 MD simulation... S8

Figure S8 Editing on GLI1 RNA by full-length hADAR3 M3 in U87 cells S9

Figure S9 Overall A-to-I editing sites identified from U87 cells expressing hADAR3 M3 or E434A S10

Figure S10 Confirmation of three randomly picked ADAR3 M3 editing sites in CDS region via Sanger sequencing S11

Table S1. Sequences for oligonucleotides used in this study S11

Table S2 – S3. List of elevated editing sites identified from U87 cells expressing hADAR3 M3 .... S13

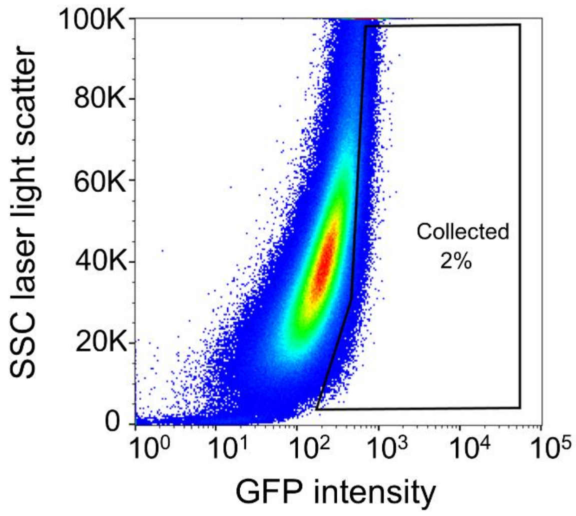

Figure S1. FACS to screen ADAR3 library covering top 11 mutations predicted by Janus.

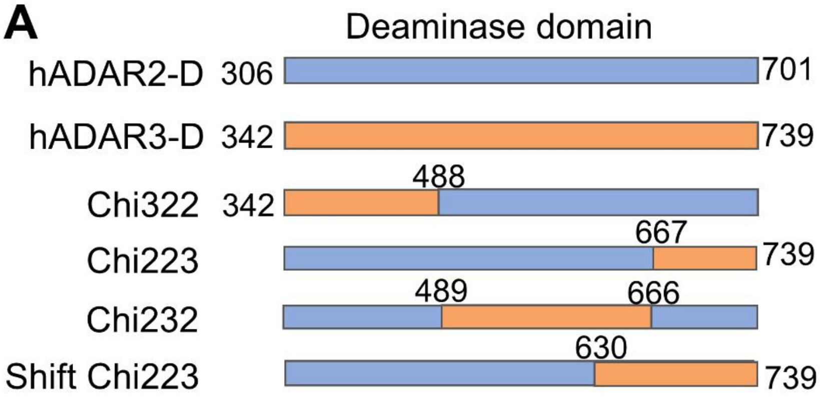

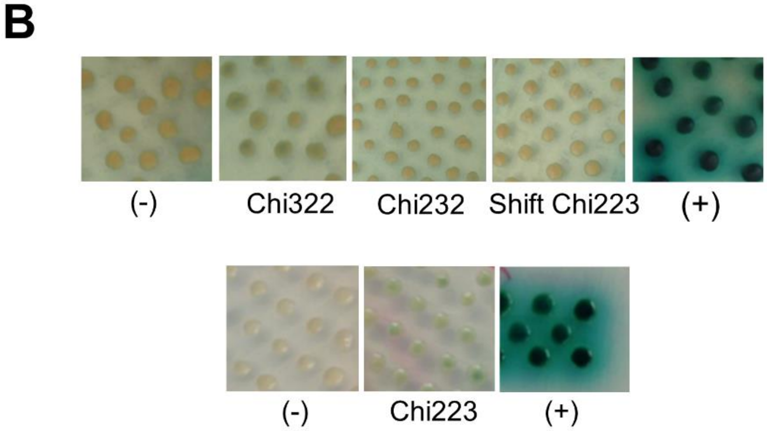

Figure S2. Colorimetric assay to evaluate activities of ADAR2-D/ADAR3-D chimeras. (**A**) Scheme of composition of hADAR2-D/hADAR3-D chimeras. The numberings in the chimeras are based on hADAR3 sequence. (**B**) Top: Yeast colonies were incubated at 30 °C for > 30 days. Bottom: Yeast colonies were incubated at 30 °C for 9 days. The (-) and (+) symbols refer to the inactive hADAR2-D E396A mutant and the wild type hADAR2-D, respectively.

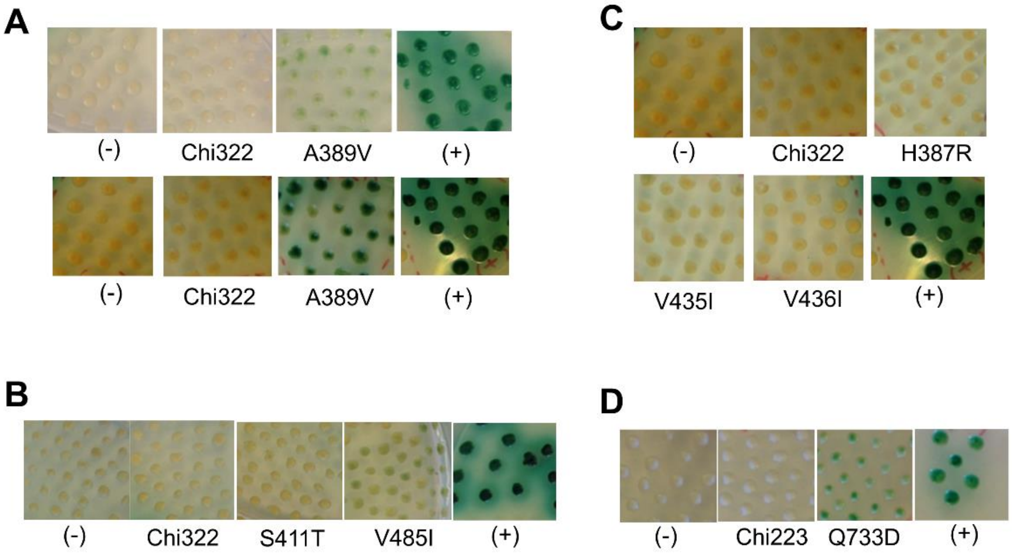

Figure S3. Colorimetric assay to evaluate effect of mutations predicted by Janus on the activities of Chi322 or Chi223. (**A**) Top: Yeast colonies were incubated at 30 °C for 10 days. Bottom: Same cultures as the top incubated at 30 °C for 28 days. (**B**) Yeast colonies were incubated at 30 °C for 21 days. (**C**) Yeast colonies were incubated at 30 °C for 28 days. (**D**) Yeast colonies were incubated at 30 °C for 4 days. In A-D, the (-) and (+) symbols refer to the inactive hADAR2-D E396A mutant and the wild type hADAR2-D, respectively.

```
hADAR3-D   342 IQMPGHAPGRARRTPMPQEFADSISQLVTQKFREVTTDLTPMHARHKALA 391
hADAR2-D   306 SRQP--IPSEGLQLHLPQVLADAVSRLVLGKFGDLTDNFSSPHARRKVLA 353
hADAR1-D   833 ------LPLTG------STFHDQIAMLSHRCFNTLTNSFQPSLLGRKILA 870

hADAR3-D   392 GIVMTKGLDARQAQVVALSSGTKCISGEHLSDQGLVVNDCHAEVVARRAF 441
hADAR2-D   354 GVVMTTGTDVKDAKVISVSTGTKCINGEYMSDRGLALNDCHAEIISRRSL 403
hADAR1-D   871 AIIMKKDSED-MGVVVSLGTGNRCVKGDSLSLKGETVNDCHAEIISRRGF 919

hADAR3-D   442 LHFLYTQLELHLSKRREDSERSIFVRLKEGG-YRLRENILFHLYVSTSPC 490
hADAR2-D   404 LRFLYTQLELYLNNK-DDQKRSIFQKSERGG-FRLKENVQFHLYISTSPC 451
hADAR1-D   920 IRFLYSELMKYNSQT---AKDSIFEPAKGGEKLQIKKTVSFHLYISTAPC 966

hADAR3-D   491 GDARLHSP--YEITTDLHSSKHL---VRKFRGHLRTKIESGEGTVPVRGP 535
hADAR2-D   452 GDARIFSP--HEPILEEPADRHP---NRKARGQLRTKIESGEGTIPVRSN 496
hADAR1-D   967 GDGALFDKSCSDRAMESTESRHYPVFENPKQGKLRTKVENGEGTIPVESS 1016

hADAR3-D   536 SAVQTWDGVLLGEQLITMSCTDKIARWNVLGLQGALLSHFVEPVYLQSIV 585
hADAR2-D   497 ASIQTWDGVLQGERLLTMSCSDKIARWNVVGIQGSLLSIFVEPIYFSSII 546
hADAR1-D  1017 DIVPTWDGIRLGERLRTMSCSDKILRWNVLGLQGALLTHFLQPIYLKSVT 1066

hADAR3-D   586 VGSLHHTGHLARVMSHRMEG-----VGQLPASYRHNRPLLSGVSDAEAR- 629
hADAR2-D   547 LGSLYHGDHLSRAMYQRISN-----IEDLPPLYTLNKPLLSGISNAEAR- 590
hADAR1-D  1067 LGYLFSQGHLTRAICCRVTRDGSAFEDGLRHPFIVNHPKVGRVSIYDSKR 1116

hADAR3-D   630 QPGKSPPFSMNWVVGS-ADLEIINATTGRRSCGGPSRLCKHVLSARWARL 678
hADAR2-D   591 QPGKAPNFSVNWTVGD-SAIEVINATTGKDELGRASRLCKHALYCRWMRV 639
hADAR1-D  1117 QSGKTKETSVNWCLADGYDLEILDGTRG--TVDGPRNELSRVSKKNIFLL 1164

hADAR3-D   679 YGRLSTRTP-SPGDTPSMYCEAKLGAHTYQSVKQQLFKAFQKAGLGTWVR 727
hADAR2-D   640 HGKVPSHLLRSKITKPNVYHESKLAAKEYQAAKARLFTAFIKAGLGAWVE 689
hADAR1-D  1165 FKKLCSFRYRRDLLRLS-YGEAKKAARDYETAKNYFKKGLKDMGYGNWIS 1213

hADAR3-D   728 KPPEQQQFLLTL-- 739
hADAR2-D   690 KPTEQDQFSLTP-- 701
hADAR1-D  1214 KPQEEKNFYLCPVL 1227
```

Figure S4. Sequence alignment between three ADARs depicting chimera construction strategy and mutations introduced in hADAR3 M3. The five mutations introduced in ADAR3 M3 are labeled with the WT amino acids in magenta and amino acids mutated to in green. Junction sites in ADAR3-D/ADAR2-D chimeras are marked with red arrows.

A

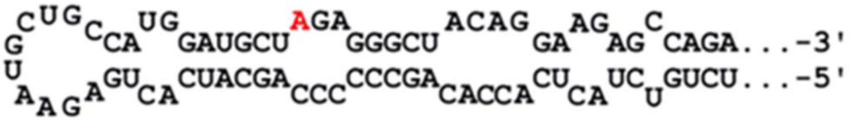

B

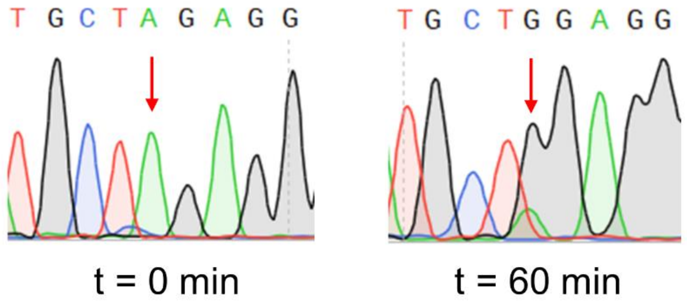

Figure S5. hADAR3-D M3 editing on human GLI1 mRNA. (**A**) the strucutre of GLI1 RNA used in the deamination reaction predicted by Mfold. (**B**) Sanger sequencing traces of editing at t = 0 min and t = 60 min. The editing site is shown by an arrow.

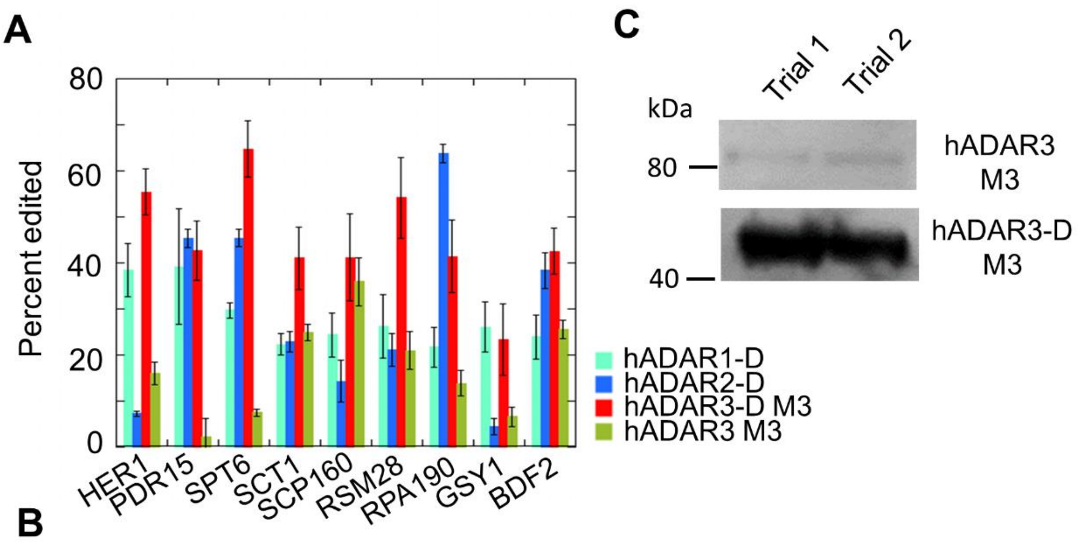

B

```
H. sapiens ADAR2           GDARIFSPHEPILEEPADRHPNRKARGQLRTK
D. melanogaster ADAR2      GDARIFSPHEND--TGVDKHPNRKARGQLRTK
T. rubripes ADAR2          GDARIFSPHEAGVEDQGDRHPNRKARGQLRTK
D. Opalescens ADAR2        GDARIFSPHGQDVET-GDRHPNRKARGQLRTK
G. gallus ADAR2            GDARIFSPHEAAQEDQGDRHPNRKARGQLRTK
H. sapiens ADAR3           GDARLHSPYEITTDLHSSKHLVRKFRGHLRTK
O. orca ADAR3              GDARLHSPYELTTDLSSSKYPARKSRGHLRTK
L. weddellii ADAR3         GDARLHSPYEITTDLNSRKHIVRKLRGHLRTK
M. p. furo ADAR3           GDARLHSPYEITADLTSSKHVVRKLRGHLRTK
C. griseus ADAR3           GDARLNSPYEITTDLNSTKHIVRKFRGHLRTK
O. Cuniculus ADAR3         GDARLNSPYEITTDLNSSKHIVRKFRGHLRTK
```

Figure S6. Editing on yeast RNA substrates by hADAR3 M3 and sequence alignment of 5' binding loops between ADAR2 and ADAR3. (**A**) hADAR1-D, hADAR2-D, hADAR3-D M3 and full-length hADAR3 M3 editing on yeast endogenous RNA. (**B**) Sequence alignment of 5' binding loops between ADAR2 and ADAR3. Green: conserved in both ADAR2 and ADAR3; blue: conserve in only ADAR2; red: conserved in only ADAR3; black: un-conserved. (**C**) Expression of full-length hADAR3 M3 (top) and hADAR3-D M3 (bottom) in yeast.

A

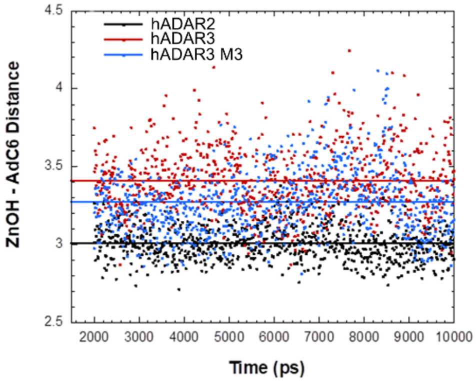

B

	hADAR2	hADAR3	hADAR3 M3
Mean	3.00	3.40	3.27
std	0.107	0.203	0.200

Figure S7. MD simulation. (**A**) MD simulation showing the distance between the zinc-coordinated hydroxyl group and the C6 position of the editing A in wild-type ADAR2, wild type ADAR3 and ADAR3 M3. (**B**) Mean distance values and standard deviation corresponding to the figure in (**A**).

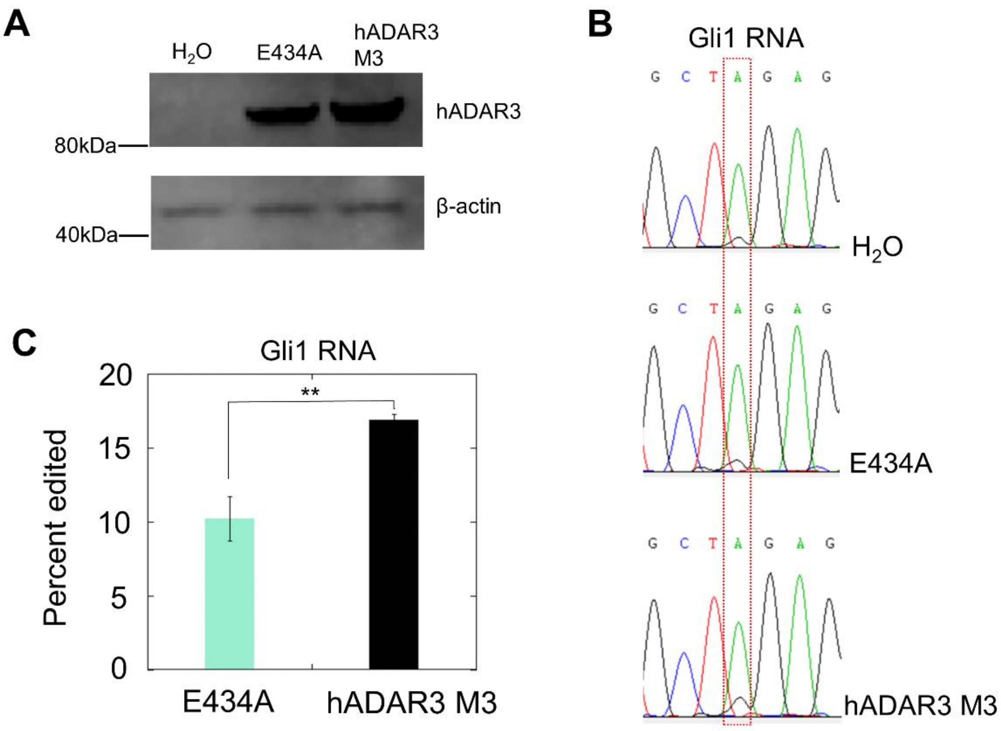

Figure S8. Editing on GLI1 RNA by full-length hADAR3 M3 in U87 cells. **(A)** Overexpression of hADAR3 in U87 cells. Cells were transfected with H_2O (left), the inactive hADAR3 mutant E434A plasmid (middle) and full-length hADAR3 M3 plasmid (right). Actin is the loading control. **(B)** Sequence traces for RT-PCR products of the edited region in GLI1 mRNA from total RNA isolated from cells transfected with H_2O, E434A or hADAR3 M3. The editing site is boxed. **(C)** Quantification of the editing at the GLI1 site shown in (**B**). Error bar indicates SD, n≥3. Student *t* test was used to determine statistical significance of difference between groups. ** means $p < 0.01$.

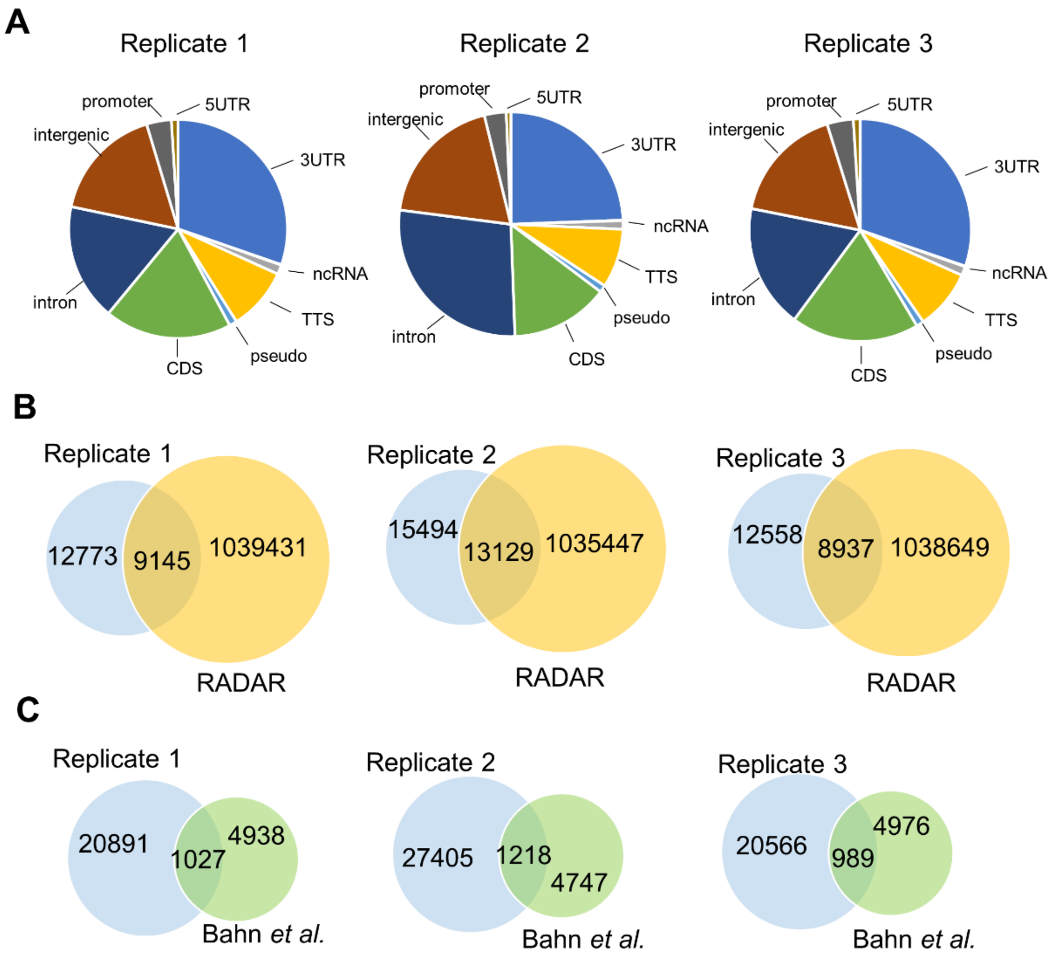

D

Replicate	**1**	**2**	**3**
Total number of A-to-I editing sites	21918	28623	21525
Number of sites in repetitive regions	12517	17746	11994
Number of sites overlapped with RADAR	9145	13129	8937
Number of sites overlapped with Bahn *et al.*	1027	1218	989

Figure S9. Overall A-to-I editing sites identified from U87 cells expressing hADAR3 M3 or E434A. (**A**) Pie chart showing the distribution of editing sites in different regions of RNA for each replicate. (**B**) Venn plot showing overlap of A-to-I editing sites identified in this study and RADAR (1). (**C**) Venn plot showing overlap of A-to-I editing sites identified in this study and sites reported by Bahn *et al.* (2). (**D**) Summarization of statistics corresponding to each replicate.

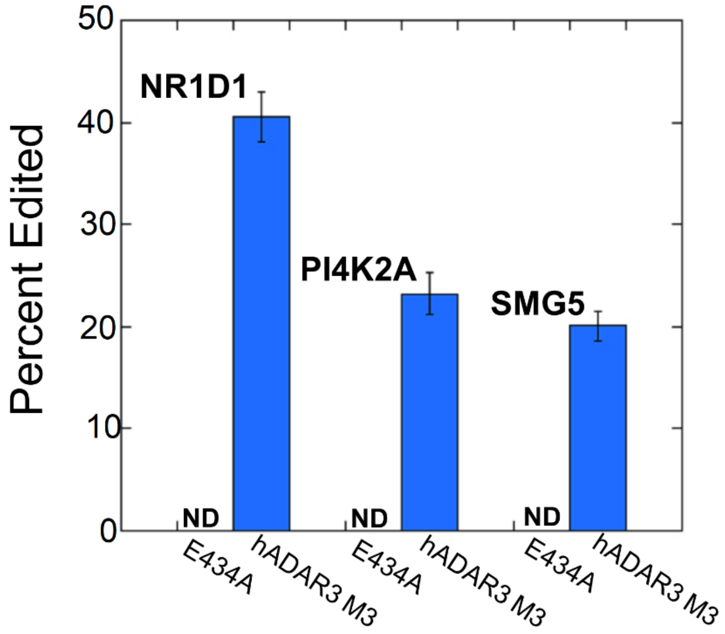

Figure S10. Confirmation of three randomly picked ADAR3 M3 editing sites in CDS region via Sanger sequencing. Error bar indicates SD, n≥3.

Table S1. Sequences for oligonucleotides used in this study.

i) Sequences for constructing ADAR2/ADAR3 chimeras.

**Name**	**Sequence**
Chi322-A3-for	5'-GCGTACGGATCCGTAACCATGTCACACC-3'
Chi322-A3-rev	5'-CATCTCCACAGGGAGAAGTAGAAACGTACAAGTGGAACAAGA-3'
Chi322-A2-for	5'-CGTTTCTACTTCTCCCTGTGGAGATGCCAGAATC-3'
Chi322-A2-rev	5'-GCGTACCTCGAGTCAGGGCGTGAGT-3'
Chi223-A2-for	5'-GCGTACGGATCCGTAACCATGTCACACC-3'
Chi223-A2-rev	5'-CAAAACGTGCTTACACAGGCGGGACGC-3'
Chi223-A3-for	5'-TCCCGCCTGTGTAAGCACGTTTTGTCTGCTAGATGG-3'
Chi223-A3-rev	5'-GCGTACCTCGAGCTACAAAGTCAACAAGAATT-3'

ii) Sequences for RT-PCR of endogenous RNAs in yeast cells.

Name	Sequence
BDF2 RT FWD	5'-GCAATGCCACCAAGAGTTTTACCCGC-3'
BDF2 RT RVS	5'-GCAATCGGATCAACAGGTTGTAAAAAGGG-3'
HER1 RT FWD	5'- AGAAGAGGAAGGTGAATTACATAAATGG-3'
HER1 RT RVS	5'-CGGTAGGAATCTTTGGTAAGATTTTTG-3'
GSY1 RT FWD	5'-AAAAGGGTATTGGCGCTGAG-3'
GSY1 RT RVS	5'-CATAATCCAAACCAAGGATAGGGT-3'
RSM28 RT FWD	5'-GCATCTAAAAGGAGAAAGGCTCC-3'
RSM28 RT RVS	5'-CCCAAATTTGGCGATCTGTAA-3'
SCP160 RT FWD	5'-AATTGGTAACAAGGGCTCCAAC-3'
SCP160 RT RVS	5'-CCAATCAGACTACCATGAACTTAACTG-3'
SCT1 RT FWD	5'-TTGAAATACCGAAGGAACTAGTCG-3'
SCT1 RT RVS	5'-TATAGAATTCGTAACCTTTGACCATTCT-3'
SPT6 RT FWD	5'-TCAACGTCACGCAGGTGC-3'
SPT6 RT RVS	5'-TGTTCTCTTTGCACGGGCT-3'
RPA190 RT FWD	5'-ACATCTAATGATGTTGCTGCTGTG-3'
RPA190 RT RVS	5'-ACAGCCTTGGTCAAGAATTGAC-3'

iii) Sequences for nested RT-PCR of endogenous GLI1, DUSP1 and EGR1 transcripts in U87 cells.

Name	Sequence
GLI1 RT FWD	5'-CGGGCAAGATATGCTTCAGC-3'
GLI1 RT RVS	5'-AGCCCAGAGTGGGAAGGG-3'
GLI1 Nest FWD	5'-GCAGGGGTCACCCGG-3'
GLI1 Nest RVS	5'-AGCCCAGAGTGGGAAGGG-3'
DUSP1 RT FWD	5'-GAGGCTCTTCACATCCCATTGGGACTCCATG-3'
DUSP1 RT RVS	5'-CAGGGGCGAGCAAAAAGAAACCGGATCACAC-3'

DUSP1 Nest FWD	5'- CTCCTTGAGAGGAGAAATGCAATAACTCTGGGAGG-3'
DUSP1 Nest RVS	5'-ACTGAGTCCTTTCTCTTCTGCCCCATTTTGTC-3'
EGR1 RT FWD	5'-TCTCAGAGCATGTGTCAGAGTGTTGTTCCG-3'
EGR1 RT RVS	5'-CTGCATGTTCATAACATACAAAAATCGCCGCC-3'
EGR1 Nest FWD	5'-TTAACCTTTTTGTAAATACTGCTTGACCGTACTCT-3'
EGR1 Nest RVS	5'-TACTCAGTAGGTAACTACAACATTCCAACTCCTGA-3'

**Table S2-S3 appear as separate excel files*

Table S2 – S3: List of elevated editing sites identified from U87 cells expressing hADAR3 M3. **Sheets 1**, **2** and **3** in **Table S2** lists elevated editing sites identified from biological replicates 1, 2 and 3, respectively. **Table S3** lists elevated editing sites appearing in all three biological replicates (**Sheet1**), in two biological replicates (**Sheet2**) and in only one biological replicate (**Sheet3**).

1. Ramaswami, G. and Li, J.B. (2014) RADAR: a rigorously annotated database of A-to-I RNA editing. *Nucleic Acids Res.*, **42**, D109-D113.
2. Bahn, J.H., Lee, J.H., Li, G., Greer, C., Peng, G. and Xiao, X. (2012) Accurate identification of A-to-I RNA editing in human by transcriptome sequencing. *Genome Res.*, **22**, 142-150.
